# Functionalization of textile using *streptomyces erythrogriseus* GH80 brown bioactive pigment with in silico studies

**DOI:** 10.1186/s12866-025-04503-5

**Published:** 2025-12-17

**Authors:** Gehad H. El Sayed, Mohamed Fadel, Rasha Fouad, Hend M. Ahmed, Ahmed A. Hamed

**Affiliations:** 1 Microbial Chemistry Department, Biotechnology Institute, National Research centre, Dokki, Giza Egypt; 2 Medicinal and Aromatic Plants Research Department, National Research Centre, Dokki, Giza Egypt; 3 Dyeing, Printing and Intermediate Auxilaries Department, Textile Research and Technology Institute, National Research Centre, Dokki, Giza Egypt

**Keywords:** *Streptomyces* sp., Pigment, GC/MS characterization, Textile printing, Antimicrobial fabrics, And molecular docking

## Abstract

In our current study, we locate, summarize, and assess the information about the potential of Streptomyces strains as biological sources of bioactive pigments. We discovered *S. erythrogriseus* GH80, a new bacterium that produces brown pigment using 16 S rRNA. Bacteria produced the brownest pigment under optimal conditions: starch 2.0% (w/v), potassium nitrate 0.2% (w/v), dibasic sodium phosphate as a phosphorous source, 2% inoculum size, pH level 8, volume ratio 20%, 180 rpm rotation speed, 37°C temperature, and 8-day incubation. Using ethanol, pigment extraction worked effectively. The data showed no significant pigment change at 40, 50, or 60°C. Increased heat treatment of pigment extract to 70, 80, 90, and 100°C for 60 min maintained pigment retention at 96, 95, 92, and 90% of total content. The pigment extract demonstrated its maximum stability at alkaline pH values of 8.0, 8.5, 9.0, and 10.0 over the holding period. Full UV-visible spectroscopic screening was utilized to characterize pigments, with a λmax at 420 nm and extract probable absorption maxima between 380 and 460 nm. GC/MS analysis of the brown pigment extract found that terbinafine (66.46%) was the main component, followed by palmitic acid, methyl ester (8.55%), 10-Octadecenoic acid, methyl ester (4.32%), and other fatty acids. The brown pigment from *S. erythrogriseus* GH80 possesses biological activity, according to GC/MS analysis. *S. erythrogriseus* strain GH80 is a viable alternative to synthetic dyes, producing brilliant colors and providing self-cleaning and UV protection. At certain concentrations and pH levels, cotton showed the most brilliant color strength. The pigment improved the self-cleaning characteristics of the dyed materials by removing stains. Outdoor uses were possible with dyed fabrics’ UV protection. Due to its fastness, the pigment may be used in sustainable textile manufacturing. Treatment of wool and polyester textile samples showed greater pathogen-fighting efficiency. *E. coli* and *P. aeruginosa* inhibited cotton 100% (67.9% and 76.16%, respectively), while *S. typhi* inhibited polyester (86.6%). *P. aeruginosa* inhibited cotton/polyester 50/50 (62.059%).

**Graphical abstract:**

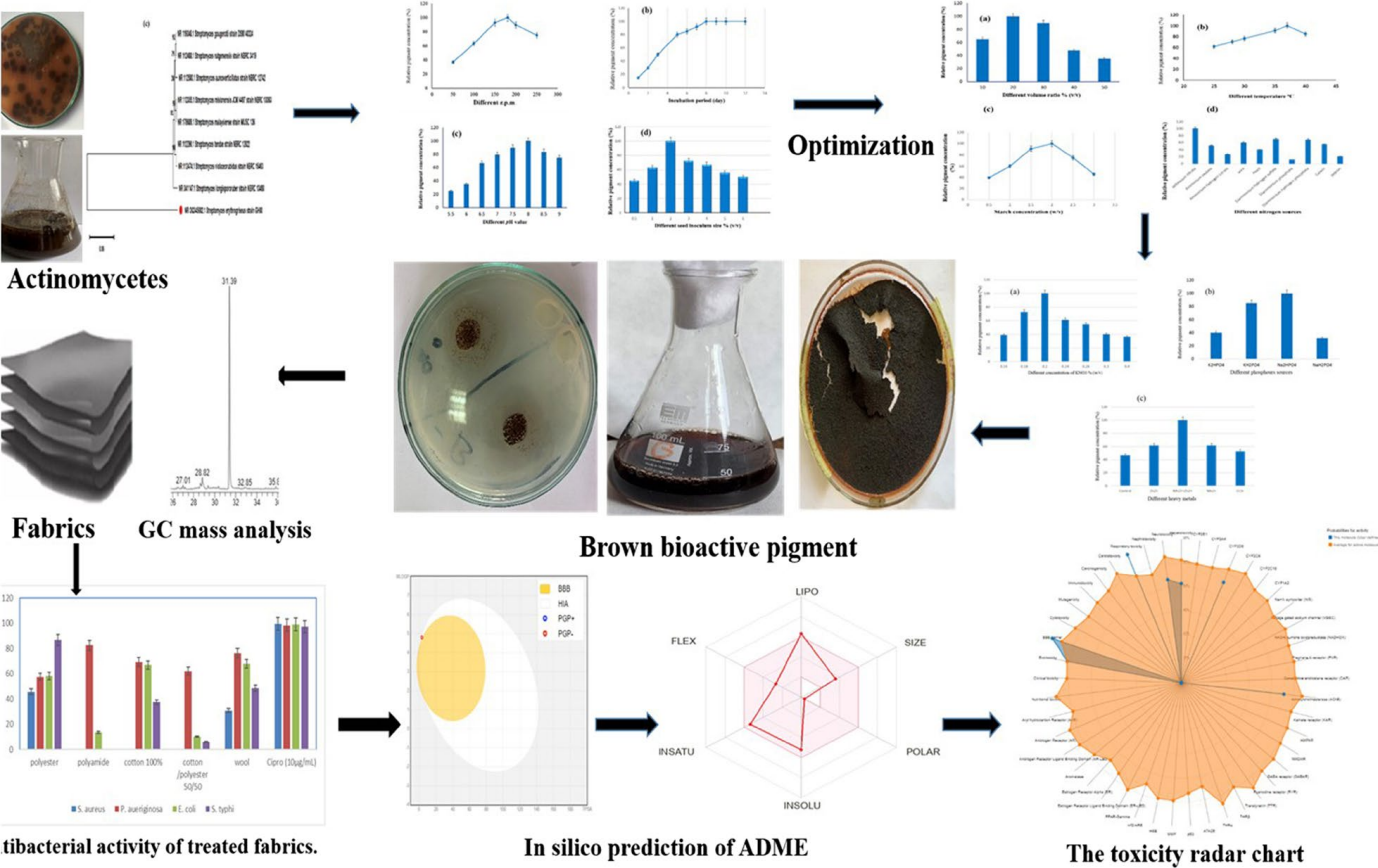

**Supplementary Information:**

The online version contains supplementary material available at 10.1186/s12866-025-04503-5.

## Introduction

The need for natural colorants has increased as a result of the negative impacts of chemically manufactured colors [1]. Additionally, there is a global trend toward using natural colorants rather than synthetic ones in the food, cosmetic, textile, and pharmaceutical industries due to negative views and close scrutiny of synthetic pigments held by numerous customers [2]. Pigments that are produced naturally are obtained from large-scale sources such as microbes, plants, algae, and insects. Plants and microorganisms prefer the biotechnological production of pigments due to its easy understanding of suitable culture techniques and processing methods [3]. Various colors, including melanins, phenazines, flavones, quinines, carotenoids, violacein, indigo, and monascins, can be produced on a large scale by filamentous fungi [4, 5]. Alves et al. (2025) had pigment production by *Pseudofusicoccum sp* [4]. Lee, CC. (2025) had novel microbial bioactive compounds [5]. Microbial pigments have many advantages, even though natural pigments can be produced from a wide range of sources, as previously mentioned [6]. The microbial production of pigments is not influenced by changes in seasons [6]. It is easily produced even on a large scale and cheap, and the high stability of its produced pigment is as stated by Díez et al. (2025) [3]. Astaxanthin, canthaxanthin, carotenoids, melanins, indigoidine, flavonoids, and quinones are examples of pigmented secondary metabolites [7, 8]. Ramya et al. (2025) had prodigiosin pigment from *Streptomyces diastaticus* [8]. These compounds have been shown to be effective and could have use in the treatment of a variety of diseases [7–9]. Vignesh et al. (2025) produce red bioactive pigment from *Streptomyces griseorubiginosus* [9]. They also possess specific characteristics as antibiotic, anticancer, and immunosuppressive agents [9]. For instance, microbial anthocyanins have a variety of biological activities, including decreasing the risk of cancer and suppressing the response of the immune system [10]. *Streptomyces* sp. is one of the most interesting genera of microbial pigments because of its high rate of reproduction and ability to produce melanin, one of the most widely used pigments in industry [11–13]. Furthermore, the intriguing genetic dispersion of these actinomycetes makes them attractive for repetition in the biotechnology sector [13–15]. Furthermore, *Streptomyces* are widely recognized for their ability to produce enormous secondary metabolites; a large number of these produced metabolites had biological activity, like cytotoxins, antibiotics, anti-inflammatory agents, and antioxidants [7–9, 16–18]. Chinnappa et al. (2024) had bioactive pigments from actinobacteria [14]. Many of the colored compounds produced by *Streptomyces* sp. could be an intriguing opportunity for producing bioactive pigments [5, 15, 19]. Mokhnache et al. (2025) obtained silver nanoparticles (AgNPs) that were green-synthesized using a red pigment produced by the *Streptomyces sp.* [15]. Additionally, many of the colored compounds produced by *Streptomyces* could be an intriguing opportunity for producing bioactive pigments, considering the bioactivity potential exhibited for *Streptomyces* strains [5, 7–9, 15, 16]. Taking into consideration that safe pigments are needed in a variety of circumstances, together with the additional useful characteristics and biotechnological potential of *Streptomyces* sp.

Girma et al. 2025 studied pigment-producing actinomycetes from Sof-Umer cave rocks and sediments [16]. Numerous factors influence biotechnological processes for producing pigments, such as pH levels and temperature of the environment, which have important effects on the formation of metabolites like pigment [17]. Daramola et al. (2025) made optimization and spectral characterization for red pigment from *Deinococcus proteolyticus* [17]. Another important factor is the culture medium’s components, which include phosphorous sources, nitrogen sources, and carbon sources. The generation of bio-pigments can be enhanced by adjusting the composition of the medium [2, 20]. This work is intended to optimize culture conditions for pigment synthesis by isolating and molecularly identifying actinomycetes producing pigment. The obtained bacteria’s pigment was then extracted, characterized, and evaluated for possible application as textile dyes for various textiles. This work is intended to optimize culture conditions for pigment synthesis by isolating and molecularly identifying actinomycetes producing pigment. The obtained bacteria’s pigment was then extracted, characterized, and evaluated for possible application as textile dyes for various textiles.

## Materials and methods

### Chemicals

All chemicals used in this study were of analytical grade. The main chemicals included PCR master mix, agarose, ethidium bromide, nutrient broth, nutrient agar, potato dextrose agar, ethyl acetate, methanol, ethanol, acetone, DMSO, ciprofloxacin, and crystal violet (all purchased from Sigma-Aldrich, USA, or Oxoid, UK).

### Sampling sites and sampling

This study has been concentrated on the soils of agricultural sources; soil samples were collected from various cultivated fields in the governorates of Assuit, Port Said, and El-Qualyubia Al-Sharqia. For five to ten minutes, the soil samples were dried at 50 to 60°C [21]. Soil samples had been obtained from agricultural fields by a sterile spatula at a depth of 0–10 cm, kept in high-density polyethylene bags that had been sterilized, and transported to the lab.

### Streptomycetes isolation

Starch nitrate agar medium was made of Solution I: twenty grams of starch with a small amount of cold distilled water was brought to a volume of 500 ml. Solution II (basal medium): KNO_3_ (2.0), K_2_HPO_4_ (1.0), CaCO_3_ (3.0), MgSO_4_ (0.5), NaCl (0.5), FeSO_4_ (0.01), and agar (20). Starch suspension and salt solutions were mixed, and pH was adjusted between 7.0 and 7.8, which was used for the isolation of actinomycetes [6]. Following the pouring plate technique, the inoculated plates were incubated at 28 °C for 7–14 days. Firm, cartilaginous, rough, chalky colonies of *Streptomyces* sp. were selected and purified.

### Preliminary screening for pigment production

Isolated actinobacteria were subcultured onto starch plates to determine that they give coloration. The colony of actinomycete that showed the required coloration was subcultured onto fresh medium by streaking until a pure culture was obtained.

### The secondary screening involved evaluating actinobacterial isolates for their pigment production capabilities

The colony of actinomycete that showed the required coloration was grown on broth starch nitrate medium to determine the most effective isolate for pigment production.

## Molecular identification of the selected isolate

### Genomic DNA extraction

According to pigment production among all isolated *Streptomyces sp.*, the *S. erythrogriseus* GH80 was chosen and genetically identified. Following the manufacturer’s instructions, genomic DNA was extracted using the Gene JET Genomic DNA Purification Kit (Thermo Scientific, USA). By using two universal primers (27 F 5′-AGAGTTTGATCCTGGCTCAG-3′; 1492R 5′-GGTTACCTTGTTACGACTT-3′), PCR amplification reactions were performed. The PCR reaction ran under the following conditions: one cycle of 95°C for 10 min, forty cycles of 95°C for 1 min, another cycle at 52°C for 1 min, another cycle at 72°C for 2 min, and a final extension of 72°C for 10 min [22]. The biomedical laboratory of Colors (Clini Lab, Egypt) performed the sequencing of the purified PCR products. Using online BLAST alignment search tools (http://www.ncbi.nlm.nih.gov/BLAST), the resultant sequences were compared with comparable known sequences found in the NCBI database in order to examine the homology and similarity of the 16 S rRNA sequences. MEGA-X software was used to create the phylogenetic trees [23–25].

### Preparation of actinomycete seed inoculum

Conical flasks, 250 ml capacity, containing 50 ml of pigment production medium were inoculated with 1% v/v of spore suspension of 5-day-old actinomycetes culture slants. Production medium composed of (g/L) starch, 20; KNO_3_, 2.0; K_2_HPO_4_, 1.0; MgSO_4_, 0.5; NaCl, 0.5; CaCO_3_, 3.0; FeSO_4,_ 0.01; pH, 7.8. Following that, incubation was carried out for five days at 30°C in a rotary shaker set to 150 rpm.

### Maximization of pigment production

Maximization of pigment production was studied by studying the effect of different culture conditions on pigment production. Different agitations of shaker speed (r.p.m.) were examined (50–250 r.p.m.), different incubation periods (1–12 days), different pH values (6, 6.5, 7, 7.5, 8, 8.5, and 9), different inoculum sizes (0.5–6%), and different broth medium volumes (10–50%) (v/v) in a 250 ml conical flask, and different incubation temperatures (25, 28, 31, 34, 37, and 40°C), as well as different concentrations of carbon sources (0.5, 1.0, 1.5, 2.0, 2.5, and 3.0) (w/v), nitrogen (potassium nitrate, ammonium oxalate, ammonium hydrogen citrate, urea, yeast extract, diammonium hydrogen sulfate, diammonium phosphate, diammonium hydrogen phosphate, casein, and peptone), and phosphorus sources (KH₂PO₄, K₂HPO₄, NaHPO₄, and Na₂HPO₄) were investigated.

Finally, different heavy metal ions (Zn^++^, Mn^++^, Cu^++^, and Cr^+++^) were examined. The production of the pigment was studied by inoculating a bacterial suspension of *S. erythrogriseus* GH80.

### Extraction and partial characterization of the brown bioactive pigment

Pigment extraction was done as described by El Sayed et al., 2023, with little difference; the solvent and filtered supernatant mixture were centrifuged for 10 min at 8,000 rpm, and the optical density of the supernatant was measured at 420 nm. The used solvents were ethanol, methanol, acetone, ethyl acetate, hexane, and water. Finally, lyophilization for the obtained pigment extract was done [6, 26, 27].

### Heat treatment effect on brown pigment stability

Temperature is another factor that affects the stability of the molecular structure of pigments, so with increasing temperature the degradation of these compounds occurs. The thermal stability of the extracted pigment was studied by heating the extracts at 40, 50, 60, 70, 80, 90, and 100°C for 60 min. Based on the absorbance values measured for brown extracts before and after heat treatments, retentions of pigment as related to heating temperature were calculated. El Sayed et al., 2023 [1, 6, 28].

### Effect of pH on brown pigment stability

The variation of the pH effect on the brown pigment stability was examined on a range of pH values of 4.0, 5.0, 6.0, 7.0, 8.0, 9.0, and 10.0 at a temperature of 37°C by measuring the absorbance values of brown pigment after one hour [1, 6, 29].

### UV/Vis spectrophotometer

The UV-visible absorption spectra of the bioactive brown pigment in solvent extracts were determined with a UV-visible spectrophotometer assay at 280–700 nm to determine the lambda maximum of the band (λ max) [26, 27].

## Biological assay

### Antimicrobial properties

An agar well diffusion assay was used to test the dried ethanol-extracted pigment as stated by El Sayed et al., 2025 [1]. Gram-negative bacteria (*Escherichia coli*), yeast (*Candida albicans*), and Gram-positive bacteria (*Bacillus cereus*,* Bacillus subtilis*,* Staphylococcus aureus*, and *Staphylococcus pyogenes*) were used in the antimicrobial activity test. Following about 24 h of bacterial growth on (NB) medium at 30°C, 100 µl of the bacterial culture was applied to the petri plates that had been prepared. By using a sterile cork borer, two 10 mm-diameter wells were made in NB agar plates. The wells were filled using 100 µl of the pigment dissolved in DMSO with a conc. of 250 µg/mL [27]. Additionally, the infected plates were left to develop for twenty-four hours. Following that, the clear zone of inhibition surrounding the inoculation well was detected and measured in mm after the inoculated plates had been allowed to develop for 24 h.

### Characterization of the pigment by GC/MS analysis

A Trace GC-TSQ mass spectrometer (Thermo Scientific, Austin, TX, USA) that had a direct capillary column TG-5MS (30 m x 0.25 mm x 0.25 μm film thickness) was used to determine the chemical composition of your samples. The temperature of the column oven was first maintained at 50°C, then raised by 5°C per minute to 250°C, kept for 2 min, and then raised by 30°C per minute to the highest temperature of 300°C, held for 2 min. Helium was employed as a carrier gas at a steady flow rate of 1 ml/min, and the injector and MS transfer line temperatures were maintained at 270 and 260°C, respectively. The autosampler AS1300 paired with GC in the split mode was used to automatically inject a diluted sample of 1 µl after a 4-minute solvent delay. In full scan mode, EI mass spectra were obtained at 70 eV ionization voltages over the m/z 50–650 range. The temperature of the ion source was fixed at 200°C. By comparing the components’ mass spectra with those from the NIST 14 and WILEY 09 mass spectral databases, the components were identified [30].

#### Using extracted brown pigment for printing on cotton, wool, and polyester textiles

The used fabrics include polyester, cotton, wool, polyamide, and cotton/polyester 50/50.

### Pretreatment of different fabrics

El-Mahala El-Kubra Company supplies the cotton fabrics in Cairo. To eliminate impurities, scrubbing the cotton fabrics for 30 min at 50 degrees Celsius in an aqueous solution with a liquor ratio of 1:50 that contained 2 g per liter of nonionic detergent solution (hospital, Clariant), they were properly rinsed in cold tap water and allowed to dry at room temperature.

Wool fabrics were cleaned for 30 min at 50°C using a solution containing 2 g/L non-ionic detergent (TERGITOLTM NP-9 Surfactant), rinsed thoroughly with water, and allowed to air dry at room temperature.

Polyester fabrics were scoured by using three grams of sodium carbonate, half a gram of wetting agent, and one gram of synthetic detergent for ten minutes.

EL-Nasr Company for Spinning, Weaving, and Dyeing, located in El-Mehalla Elkubra, Egypt, provided the bleached polyester/cotton fabric 50/50. Usually kept between 60 and 80°C (140 and 176°F), the blended fabrics were scoured in a 1:50 scouring liquid ratio with 2 g/l of nonionic detergent solution (hospital, Clariant) and an alkali agent (sodium carbonate, 1 g/l). It was then thoroughly cleaned with cold tap water and left to dry at room temperature in order to assist in lifting and removing contaminants and improve the cleaning process.

El-Nasr Spinning Weaving and Knitting Company (Shourbagy, Cairo, Egypt) provided the 210 denier/35 filament yarns for the polyamide 6 knitted fabric, which had a density of 1.14 g/cm³ and a chemical structure of NH₂—[(CH₂)₅—CONH] n—COOH. After 30 min of 60°C soaping, the fabric was carefully cleaned and allowed to air dry at room temperature.

### Used chemicals

Laboratory-grade chemicals are used to supply ammonium persulfate (NH4)_2_S_2_O_8_, urea, tannic acid, and nonionic detergent as thermal initiators. Sigma Aldrich supplied the thermal curing thickeners and binders.

## Methods

**Table:** Preparation of printing paste

The printing paste recipe	
Synthetic thickener	2 g
Binder	5-20 g
Urea	4 g
Sodium dihydrogen phosphate	0.5 g
Dyes	2 g
Water	X g
	100 g

After printing fabrics, the samples are fixed by using thermofixed for three minutes at 180°C. All of the printed samples were scoured by rinsing them completely with cold water, then washing them with hot water at 60°C using 2 g/l nonionic detergent, washing them with hot water, and finally rinsing them with cold water. The samples were then evaluated for color strength after being dried [1].

### Determination of Color Assessment and Fastness Properties

The color change of the tested sensor wool textiles was examined using tinctorial strength (K/S) and CIE Lab using an Ultra Scan PRO spectrophotometer coupled with a 10° standard viewer and D65 illuminant (Hunter Lab, USA). Blackness (0) to whiteness (100) is represented by L*, greenness (-) to redness (+) by a*, and blueness (-) to yellowness (+) by b*. AATCC Test Method (8-2016) for rubbing [31], AATCC Test Method (61-2013) for washing [32], AATCC Test Method (15-2013) [33] for sweat, and AATCC Test Method (16.1-2014) for light [34] were used to record the colorfastness qualities.

### Measuring Self-Cleaning

The following formula was used to determine the properties of the self-cleaning of treated samples': Decomposition is equal to [(K/S) s – (K/S) w] / [(K/S) s – (K/S) 0] x 100. In this case, (K/S) o represents the color strength of the unstained fabric, (K/S) s represents the color strength of the coffee-stained fabric, and (K/S) W represents the color strength of the coffee-stained fabric following light irradiation [35].

### Detection of UV protection

The Australian/New Zealand standard (AS/NZS 4366-1996) was used to calculate the UV protection factor (UPF) of the treated and untreated samples. UPF received the following ratings: excellent (UPF>40), very good (UPF: 25–39), and good (UPF: 15–24) UV protection [1].

### Statistical analysis 

To make sure the experimental data was reliable and reproducible, statistical analysis was carried out by measuring the standard deviation (SD) of triplicate measurements

## Results and discussion

### Streptomycetes isolation

*Streptomyces* sp. are usually characterized by round, convex-shaped colonies, and moreover, their rooting growth into the medium. Based on these characteristics, seventy-five actinomycete colonies were chosen and isolated. Pansomsuay et al. (2025) had *Streptomyces marinisediminis* isolated from Thai marine sediment [7]. Vignesh et al. (2025) isolated *Streptomyces griseorubiginosus* [9].

#### Preliminary screening

Out of seventy-five actinomycete colonies, three isolates had the ability to grow and give pigment (Table 1).

**Table 1:** Morphological characteristics of actionobacterial isolates having pigmentation ability

Isolate number	Spore mass	Substrate mycelium	Diffusible pigment
GH11	Grey	Red	Red
GH75	Grey	Colorless	Yellow
GH80	Grey	Dark brown	Brown

### Secondary screening

**Fig. 1:**
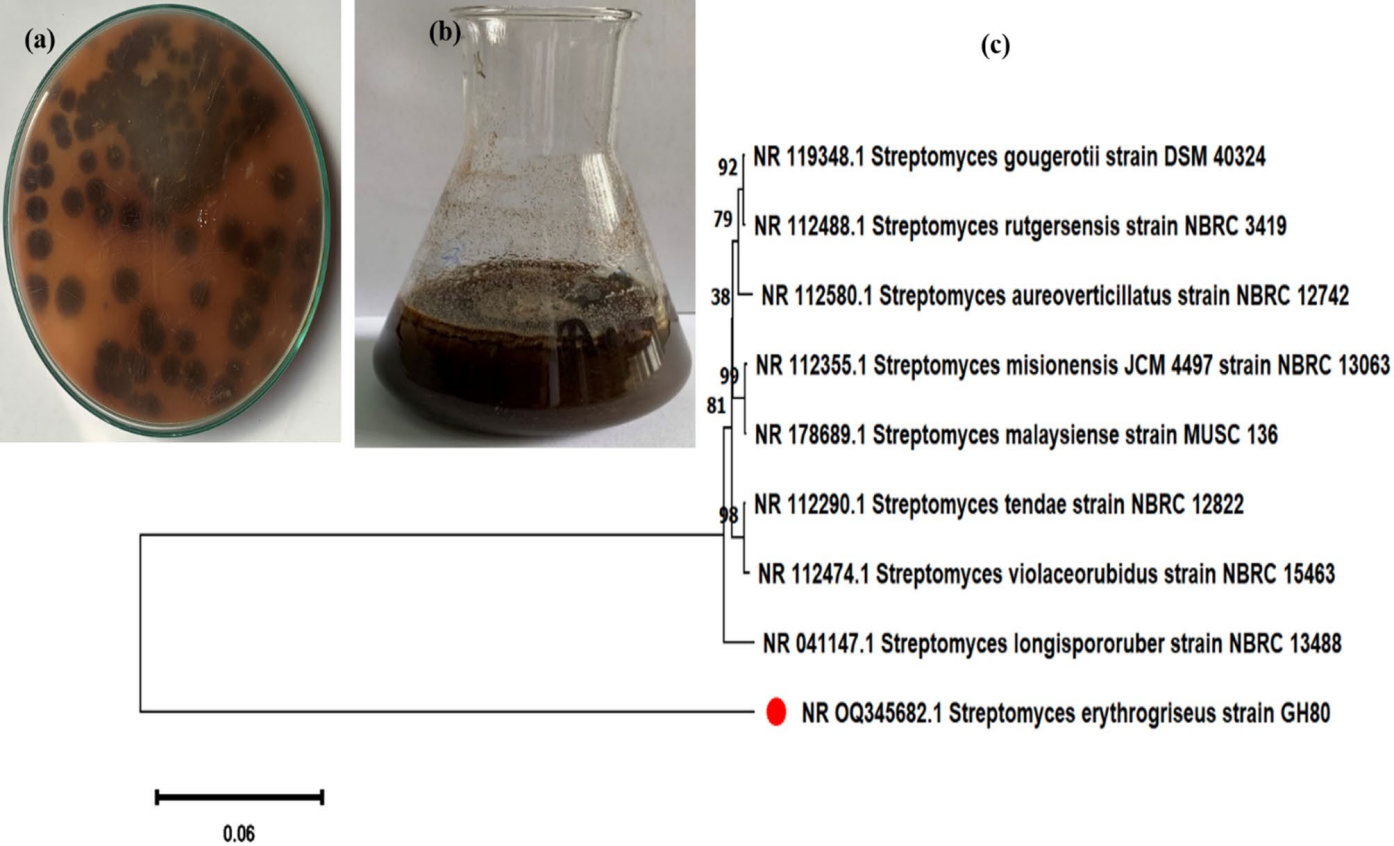
**a** Brown pigment secreted by *S. erthrogrseus* GH80 in solid media, **b** Diffusible brown pigment produced by *S. erthrogrseus* GH80 in liquid media, **c** Phylogenetic tree of *S. erythrogriseus* GH80 based on 16S rRNA gene sequences

The *S. erythrogriseus* GH80 strain was selected for further study after being discovered to be a strong generator of brown pigment (Figs. 1a and b).

### Molecular identification of the actinomycetes isolate

The 16 S rRNA sequence of the *S. erythrogriseus* GH80 was obtained and submitted to the Gene Bank database. This submitted nucleotide sequence was provided a Gene Bank accession number OQ345682.1. Based on the results of the BLAST search using the sequence data, the *Streptomyces sp.* isolate was found to be strongly related to *Streptomyces sp.* The cluster showed a close relationship in the same clade, with a bootstrap value of 100%, as presented in the cluster (Fig. 1c).

### Optimum culture conditions for highest pigment production

Optimizing cultivation conditions, such as the medium, temperature, incubation time, pH, and agitation and aeration conditions, along with selecting appropriate extraction conditions, including the solvent and method, is essential for maximizing the yield of actinomycete-derived pigments.

These factors must be carefully considered for each species to achieve the desired outcomes when pursuing biotechnological applications. The pigment produced is influenced by the species of actinomycetes, as different strains possess distinct metabolic pathways and capabilities. These processes are influenced not only by the species but also by the composition of the cultivation medium, as variations in media can influence their metabolism (carbon source, nitrogen source, and C/N ratio). The use of specialized media or additives like calcium chloride, magnesium sulfate, or iron sulfate is often employed to enhance pigment production, as stated by Diez et al., 2025 [3].

Usually, microbial pigment production is related to the growth of cells and is impacted by external factors (pH, temperature, and shaker speed), microbiological characteristics (such as spore, seed, and inoculum ages), and nutritional impacts (such as carbon and nitrogen sources) [6, 36].

### Effect of agitation (shaker speed) on brown pigment production

**Fig. 2:**
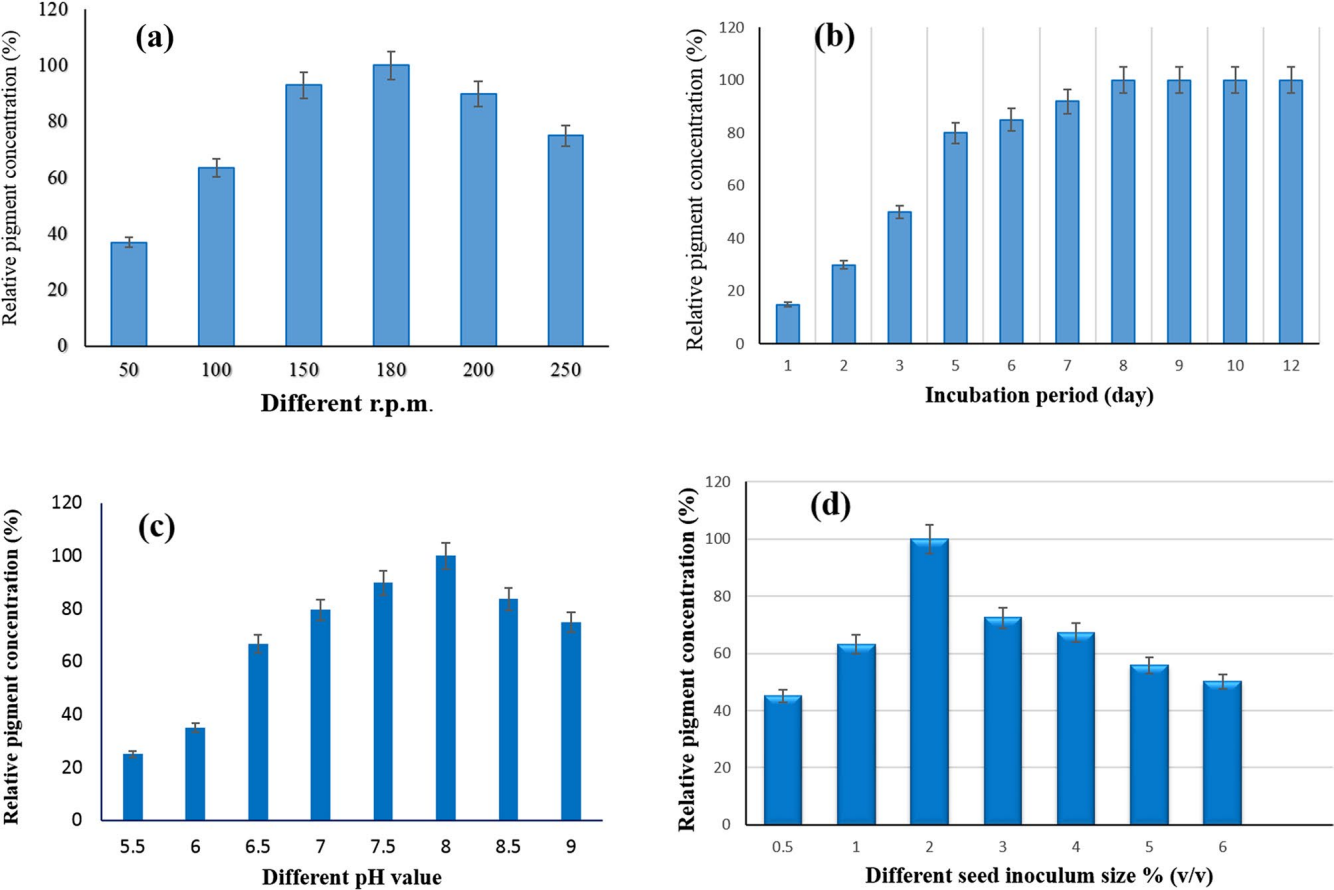
**a** Effect of Shaking speed, **b** Effect of incubation period, **c** Effect of different initial pH, **d** Effect of inoculums size % (v/v) on the brown pigment production by *S. erythrogriseus.* Data are expressed as Mean ± S.D. for *n* = 3

The findings show that the maximum bacterial growth and concentration of brown pigment are obtained from shaken cultures at 180 r.p.m. (Fig. 2a). This might be due to aeration improving the transport of oxygen and substrates, which in turn improved the growth and functionality of microbial cells [37, 38]. This result differs from El Sayed et al. (2025), who had maximum red pigment production at 150 rpm [1]. Using yeasts (*Rhodorula* sp. and *Phaffia* sp.), the same outcome was seen after incubation at an agitation rate of 180–900 rpm [39].

### The impact of incubation time on the production of brown bio-pigment by ***S.****erythrogriseus* GH80

As the fermentation time was prolonged until the eighth day, when a steady state was achieved, it became clear from confirming Fig. 2b that the density of brown pigment released by *S. erythrogriseus* GH80 in the production medium increased. El Sayed et al. (2023) and El Sayed et al. (2025) had maximum pigment production at the ninth day of the incubation period [1, 6]. It has been shown in earlier research that different strains have different ideal incubation times for producing the most pigment content. El Sayed et al. (2023) had green pigment produced by *S. nigra* strain GH12 at pH 8 [6]. After six days, *Penicillium* sp. produced more red pigment [2]. After 12 days of incubation, the maximum amount of pigment was obtained by *Penicillium purpurogenum* DPUA 1275 [40]. However, after 24 days of incubation, *Talaromyces verruculosus* produced the maximum pigment [41].

### Effect of different pH values on the production of brown bio-pigment

The pH can affect the pigment production and its shade. The color of the pigment of the same organisms produced may change depending on the pH of the production medium. Results indicated that the concentration of brown pigment produced by the *S. erythrogriseus* GH80 strain is highly influenced at high pH values, and its maximum pigment content is at pH 8 (Fig. 2c). A previous study had pH 8 as an optimum for red pigment production by bacterium isolate Mif41 [42]. The highest biomass and pigment synthesis by Penicillium sp. were noted at an initial pH of 9.0 [43]. We agree with El Sayed et al., 2025, that red pigment was produced by the *S. phaeolivaceus* strain GH27 at pH 8 [1]. Also, El Sayed et al. (2023) had green pigment produced by *S. nigra* strain GH12 at pH 8 [6].

### Impact of the size of the inoculum (v/v) on the brown bio-pigment production

Results showed that 2% (v/v) inoculum used for the medium inoculation had recorded the highest brown pigment production by *S. erythrogriseus* GH80. By using lower or higher inoculum sizes, pigment content decreased (Fig. 2 d). The same result was stated in other studies [44, 45]. El Sayed et al. (2023) had green pigment produced by *S. nigra* strain GH12 with an inoculum size of 6% (v/v) [6]. El Sayed et al. (2025) observed the production of red pigment at an inoculum size of 5% (v/v) [1]. It was reported that high inoculum size led to increased biomass and decreased pigment production due to the inhibition of the utilization of essential nutrients of the culture medium by increased biomass [46].

### The effect of medium volume on brown bio-pigment production

**Fig. 3:**
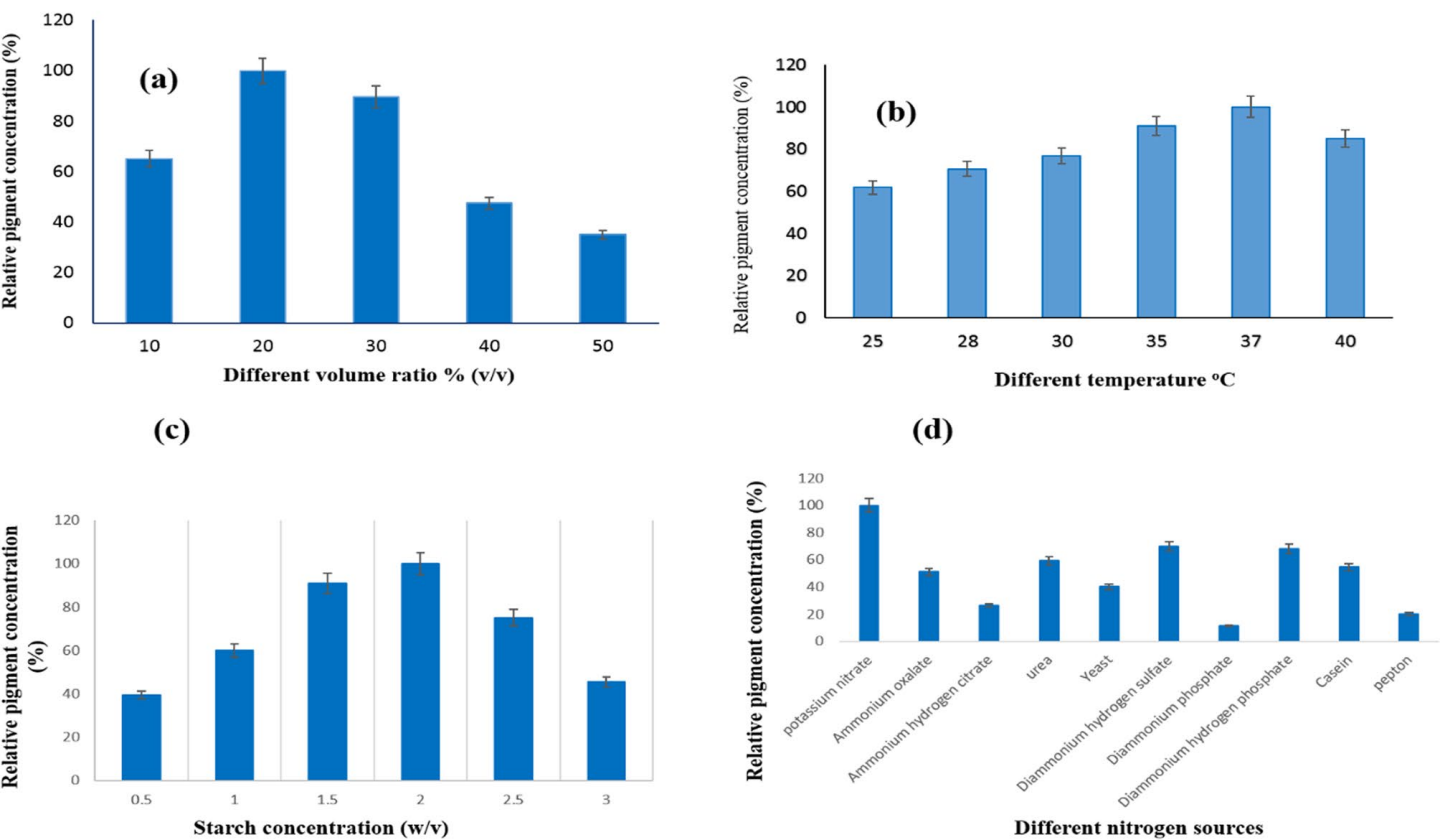
**a** Different volume ratio (v/v), **b** Different temperature ^o^C, **c** Different starch concentration (w/v), **d** Different nitrogen sources on brown pigment production by *S. erythrogriseus* GH80. Data are expressed as Mean ± S.D. for *n* = 3

Different working volumes of medium were loaded into the flasks at levels ranging from 10 to 60% and introduced in 250-ml conical flask capacity. The maximum yield was obtained at a volume ratio of 20% (v/v). The same result was obtained by El Sayed et al. (2025), who found that red pigment was produced by the *S. phaeolivaceus* strain GH27 with a medium volume of 20% (v/v) [1]. On the other hand, El Sayed et al. (2023) had green pigment produced by *S. nigra* strain GH12 with a medium volume of 75 ml [6]. Any drift of this working volume led to a significant decrease in pigment production, as described in Fig. 3a. By increasing the culture medium volume, there is a decrease in cell growth and production of pigment due to lowering oxygen content [44].

### Different incubation temperatures impact the brown bio-pigment production

Incubation temperature is one of the main factors that depends on the microorganism type. It is demonstrated that an incubation temperature of 37°C produced the highest concentration of brown pigment secreted in the growth medium by the *S. erythrogriseus* GH80 strain (Fig. 3b). The pigment was adversely affected above 37°C. Similar result: 37°C was the best temperature for green pigment production in a previous study [6]. According to studies by Joshi et al. (2011), 35°C is the ideal temperature for producing pigment [47]. It was stated that *Pseudomonas* needs 35–36°C for growth and pigment production [48], while *Monascus* sp. needs 25–28°C for pigment production [49]. El Sayed et al. (2025) had a similar result: red pigment was produced by the *S. phaeolivaceus* strain GH27 at 37°C [1].

### Impact of different starch concentrations on *S. erythrogriseus* GH80’s ability to produce brown pigment

By increasing the amount of starch added to the growth medium from 0.5% to 2.0% (w/v), the brown pigment secretion in the medium inoculated with *S. erythrogriseus* GH80 was enhanced. However, the increase in starch had a negative effect on pigment production in the medium (Fig. 3c). It is known that starch is more frequently used as a carbon source for pigment production by actinomycetes, whereas *Sarcina* sp. and *Exiguobacterium aurantiacum* FH have been shown to use glucose and fructose as more appropriate carbon sources [47, 50]. El Sayed et al. (2023) had green pigment produced by *S. nigra* strain GH12 with a starch concentration of 2.5% (w/v) [6]. El Sayed et al. (2025) had red pigment by the *S. phaeolivaceus* strain GH27 at a starch concentration of 1% (w/v) [1].

### The impact of different nitrogen sources affects the brown pigment production

For maximum pigment production, different microbes may prefer different types of nitrogenous compounds, both organic and inorganic [51]. For increased brown pigment secretion in the growth medium inoculated with *S. erythrogriseus* GH80, potassium nitrate is a better option than other inorganic nitrogen sources (Fig. 3 d). We agree with previous research on this outcome, having the highest pigment production by *Sarcina* sp. using potassium nitrate [47, 52]. On the other hand, potassium nitrate is the least effective source of nitrogen for Monascus pigment [49]. El Sayed et al. (2023) had green pigment produced by *S. nigra* strain GH12 by using ammonium nitrate as a nitrogen source [6]. El Sayed et al. (2025) had red pigment by the *S. phaeolivaceus* strain GH27 by using diammonium citrate as a nitrogen source [1].

### Effect of different concentrations of potassium nitrate on the brown bio-pigment production

**Fig. 4:**
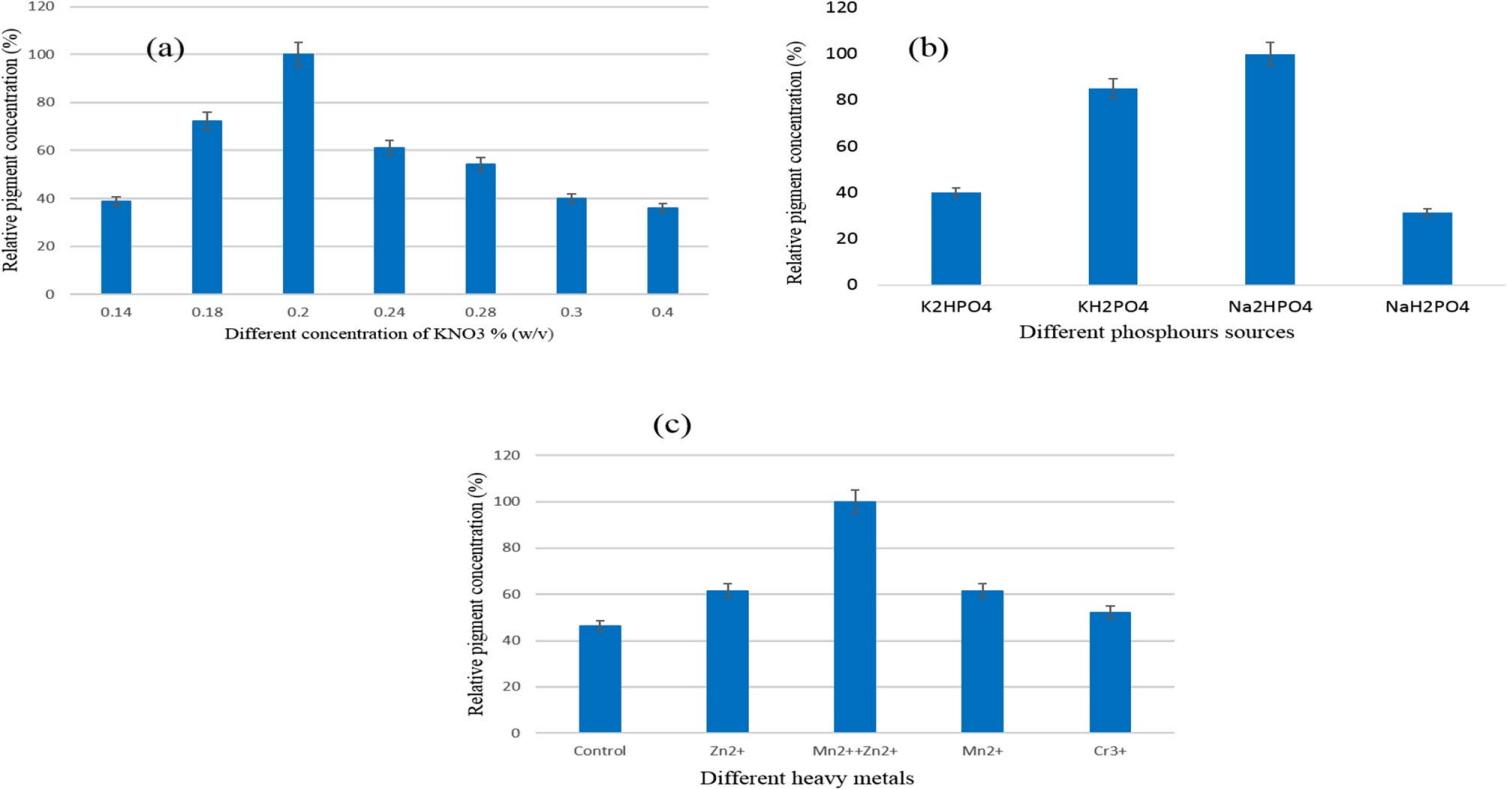
**a** Different potassium nitrate concentrations effect, **b** Different phosphorous sources effect, and **c** Different metal ions effect on brown pigment production by *S. erythrogriseus* GH80. Data are expressed as Mean ± S.D. for *n* = 3

The secretion of brown pigment in the growth medium inoculated with *S. erythrogriseus* GH80 was demonstrated in Fig. 4a. It appears that the optimal concentration of potassium nitrate to produce a high amount of brown pigment is 0.2% (w/v); concentration levels above or below this value resulted in a decrease in pigment density production. Additionally, it was discovered that potassium nitrate (0.5%) was the best nitrogen source for *Pseudomonas* sp. pigment production [52]. El Sayed et al. (2023) had green pigment produced by the *S.nigra* strain GH12 by using an ammonium nitrate concentration of 2 g/l as a nitrogen source [6].

### Impact of different phosphorus sources affect the brown pigment production

Dibasic sodium phosphate was the best source of phosphorus among the studied phosphorus sources to obtain the optimal amount of brown pigment by *S. erythrogriseus* GH80 in the growth medium. The lowest phosphorous source for pigment secretion was sodium dihydrogen phosphate (Fig. 4b). Dipotassium hydrogen phosphate was the best phosphorous source for *Streptomyces tunisiensis* W4MT573222 to produce pigment in an optimized starch-based medium [3]. This result agrees with El Sayed et al., 2025, that had dibasic sodium phosphate as a phosphorus source for the red pigment produced by the *S. phaeolivaceus* strain GH27 at pH 8 [1]. Also, El Sayed et al. (2023) had green pigment production by the *S. nigra* strain by using dibasic sodium phosphate as a phosphorus source [6].

### Different effects of metal ion addition

Minerals have an essential role in producing pigment. Zn (2 × 10^− 3^ M and 3 × 10^− 3^ M) inhibited the growth in liquid medium [49]. Inorganic salts such as Fe^2+^, Ca^2+^, Zn^2+^, Cu^2+^, Mg^2+^, and Mn^2+^ are important for *Monascus* growth in SmF; this is because they promote cell growth and MK synthesis of *Monascus* in SmF [48–50]. (Mn^2+^+ Zn^2+^) ions are very essential in the culture medium for brown pigment production by *S. erythrogriseus* GH80 (Fig. 4c). El Sayed et al., 2025, had Cu^2+^ for maximum red pigment production by the *S. phaeolivaceus* strain GH27 [1]. Also, El Sayed et al. (2023) had maximum green pigment production by the *S. nigra* strain by using Cr^+++^ metal ion [6].

### The brown bio-pigment’s extraction

**Fig. 5:**
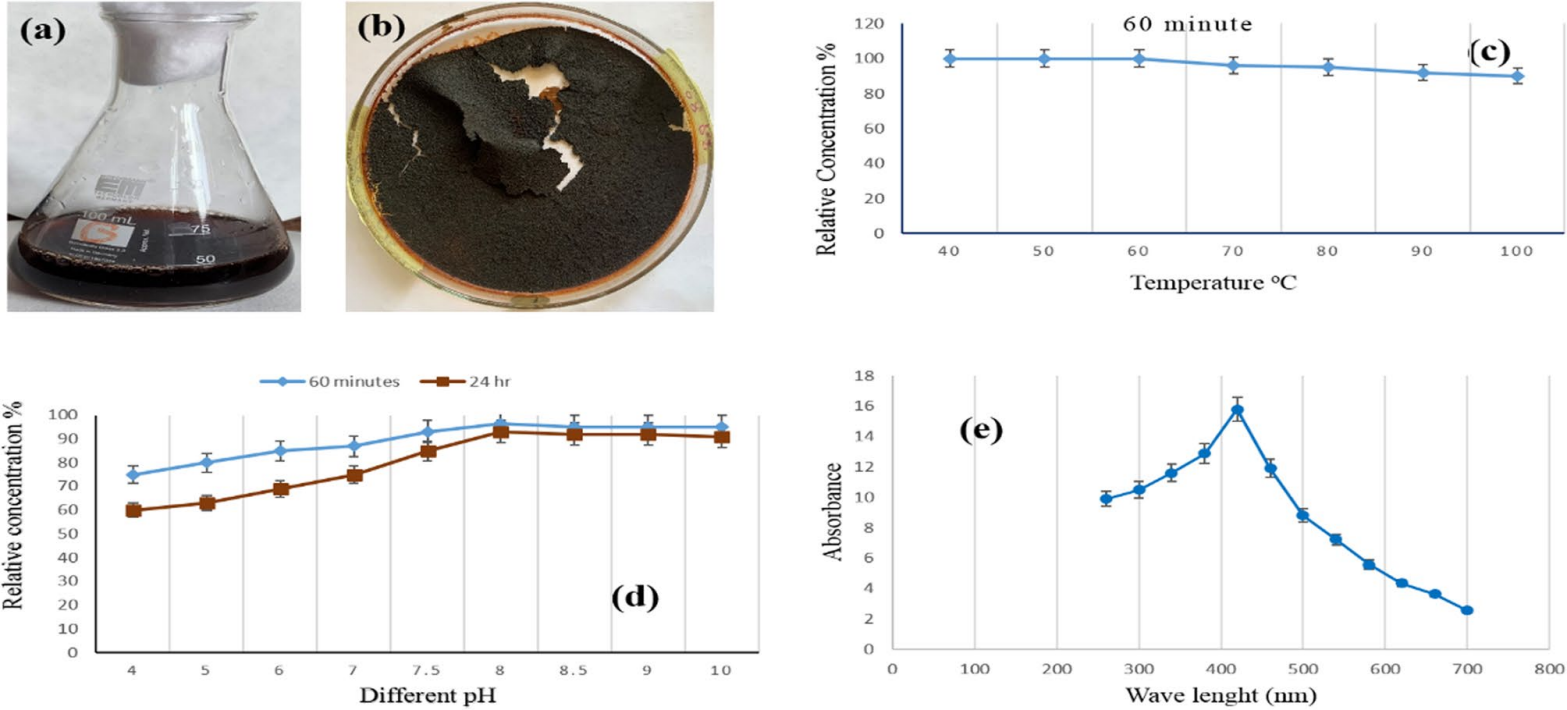
**a** Pigment extracted by ethanol solvent, **b** dried pigment after ethanol extraction, **c** heat treatment effect on brown pigment stability, **d** effect of pH on retention of brown pigment, (d) the UV absorption spectrum of extracted brown pigment. Data are expressed as Mean ± S.D. for *n* = 3

It is stated that the choice of organic solvent had a direct effect on the chemical composition of the extracted pigment [44]. In actuality, the liquid-liquid extraction technique employing ethanol as an extracting solvent yields the highest-yield brown pigment extract [51]. Following that, a lower pressure vacuum was used to dry the recovered extract (Figs. 5a and b). In other studies, methanol was identified as the optimal solvent for pigment extraction [27]. This result agreed with El Sayed et al. (2025) and El Sayed et al. (2023), who had ethanol as the best solvent for pigment production [1, 6].

### Heat treatment effect on brown bio-pigment stability

The brown pigment stability is influenced by heat treatment (Fig. 5c). No significant effect on the pigment content was done at 40, 50, and 60 °C after 60 min; retention remained high by increasing the heating temperature to 70, 80, 90, and 100 °C, respectively, and became 96, 95, 92, and 90%. This result showed retention of brown pigment extracted from *S. erythrogriseus* GH80 remained high at higher temperatures for one hour.

### Effect of pH on brown bio-pigment stability

According to the data, brown pigment was extremely stable in the case of aqueous conditions. After twenty-four hours, it was found that the brown pigment extracts remained most stable at pH 8.0 to pH 10.0 (Fig. 5 d). Similar results were by El Sayed et al. (2023). Also, El Sayed et al. (2025) had its high stability of produced red pigment at alkaline pH.

### Brown bio-pigment UV-absorption spectrum

Figure 5e displays the UV absorption spectra of the brown pigment extract produced by *S. erythrogriseus* GH80. The results showed that the brown pigment extract’s absorption maximum peak had a λmax at 420 nm and varied from 380 to 460 nm. El Sayed et al. (2023) had a λmax at 340 nm of produced green pigment. Bhat and Marar (2015) isolated orange pigment from *Salinicoccus* sp. M KJ997975 and measured its λmax at 450 nm [45].

### Antimicrobial properties of extracted brown pigment

**Fig. 6:**
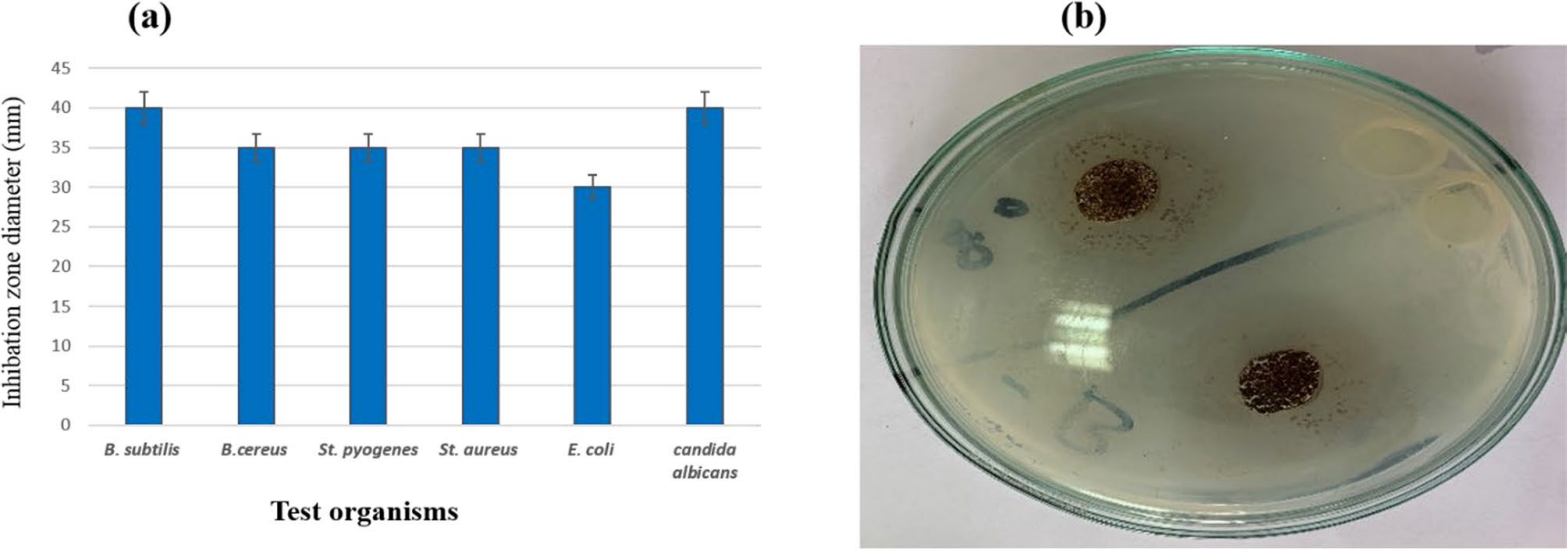
Antimicrobial activity of extracted brown pigment produced by *S. erythrogriseus* GH80

With distinct inhibitory zones that range from 30 mm for *Escherichia coli (E. coli)*, 35 mm for *Bacillus cereus (B. cereus)*, *Staphylococcus pyogenes (St. pyogenes)*, and *Staphylococcus aureus (St. aureus)*, and 40 mm for *Bacillus subtilis* (*B*. *subtilis*) and *Candida albicans*, the extracted brown pigment exhibits wide antimicrobial action. (Fig. 6). Microbial pigments additionally have distinctive biological properties, including antibacterial, antioxidant, anti-aging, photoprotection, anticancer activity, anti-inflammatory, cardiovascular disease prevention, anti-obesity, and antidiabetic activities, according to Lee et al. (2025). Vignesh et al. (2025) produce red bioactive pigment from *S. griseorubiginosus* [9].

### GC/MS characterization of brown pigment produced by ***S. erythrogriseus*** GH80

Various chromatographic techniques, such as (GC-MS), have made it possible to examine and identify metabolic pathways and their by-products with both endogenous and exogenous origins in recent times [53]. GC-MS is an analytical technique for identifying various substances in a test sample by combining the features of mass spectrometry and gas chromatography. In a previous study, 57 compounds were identified in the silylated derivative form of the dark green pigment from *S. nigra* GH12 by using GC-MS [6]. The main components were as follows: lactic acid (19.72%), methylbutanoic acid (11.82%), carbamic acid (7.80%), ethylene (6.35%), mesoerythritol (4.98%), methylpropanoic acid (4.54%), palmitic acid (2.54%), and 4-hydroxy-N-valeric acid (2.20%).

**Fig. 7:**
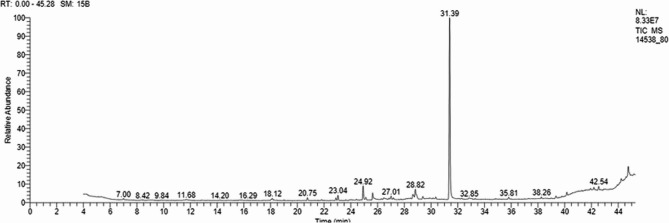
GC-MS Chromatogram of the total brown pigment of*S. erythrogriseus* GH80

The pigment analysis by GC-MS in this study yielded several components, as shown in Table 2; Fig. 7. Twelve compounds were identified and accounted for approximately 88.79%, while unidentified compounds accounted for 11.21%. It can be noticed that terbinafine was the main component (66.46%), followed by palmitic acid, methyl ester (8.55%), then 10-Octadecenoic acid, methyl ester (3.25%), 7,10-Octadecadienoic acid, methyl ester (1.88%), tetradecanoic acid, 12-methyl-, methyl ester (1.78%), hexadecanoic acid, 14-methyl-, methyl ester (1.20%), methyl 9,9-Dideutero-Octadecanoate (1.07%), stearic acid, methyl ester (1.05%), and myristic acid, methyl ester (1.03%). Colour extract GC-MS analysis showed a complex chemical profile dominated by bioactive components. Terbinafine (66.46%), a well-known antifungal, was the largest constituent and may contribute to the pigment’s antibacterial activity. Fatty acid methyl esters, such as palmitic acid (8.55%); 10-octadecenoic acid (4.32%); and stearic acid (1.05%); are known to have antimicrobial, antioxidant, and anticancer properties, indicating the extract’s biological relevance. Minor ingredients like 7,10-octadecadienoic acid methyl ester (1.88%); and tetradecanoic acid, 12-methyl-, methyl ester (1.78%) may combine to boost pigment bioactivity. The detected chemicals made up 88.79% of the total composition, leaving 11.21% as undiscovered metabolites that may contribute to biological potential.

**Table 2:** The main constituents and their relative percentages as identified with GC-MS of the main constituents of brown pigment produced by S. *erthrogrseus *GH80

R.T.	Area %	MF	MW	Compounds
18.13	0.69	C_15_H_24_O	220	Caryophyllene oxide
20.75	1.03	C_15_H_30_O_2_	242	Myristic acid, methyl ester
22.88	0.96	C_16_H_32_O_2_	256	Methyl 13-methyltetradecanoate
23.04	1.78	C_16_H_32_O_2_	256	Tetradecanoic acid, 12-methyl-, methyl ester
25.11	0.87	C_17_H_32_O_2_	268	Palmitoleic acid, methyl ester
25.63	8.55	C_17_H_34_O_2_	270	Palmitic acid, methyl ester
27.01	1.20	C_18_H_36_O_2_	284	Hexadecanoic acid, 14-methyl-, methyl ester
28.64	1.88	C_19_H_34_O_2_	294	7,10-Octadecadienoic acid, methyl ester
28.82	3.25	C_19_H_36_O_2_	296	10-Octadecenoic acid, methyl ester
28.92	1.07	C_19_H_36_D_2_O_2_	300	Methyl 9,9-Dideutero-Octadecanoate
29.38	1.05	C_19_H_38_O_2_	298	Stearic acid, methyl ester
31.39	66.46	C_21_H_25_N	291	Terbinafine

It could be concluded that the pigment contains mainly terbinafine, several fatty acids, and one oxygenated sesquiterpene (caryophyllene oxide). It is well known that terbinafine (C_21_H_25_N) is an allylamine fungicidal agent effective against dermatophytes and some yeasts, used to treat superficial fungal infections of the skin and nails [54, 55].

## Printing of cotton, wool, and polyester fabrics with extracted brown pigment

### The effect of pH on the printing paste

**Fig. 8:**
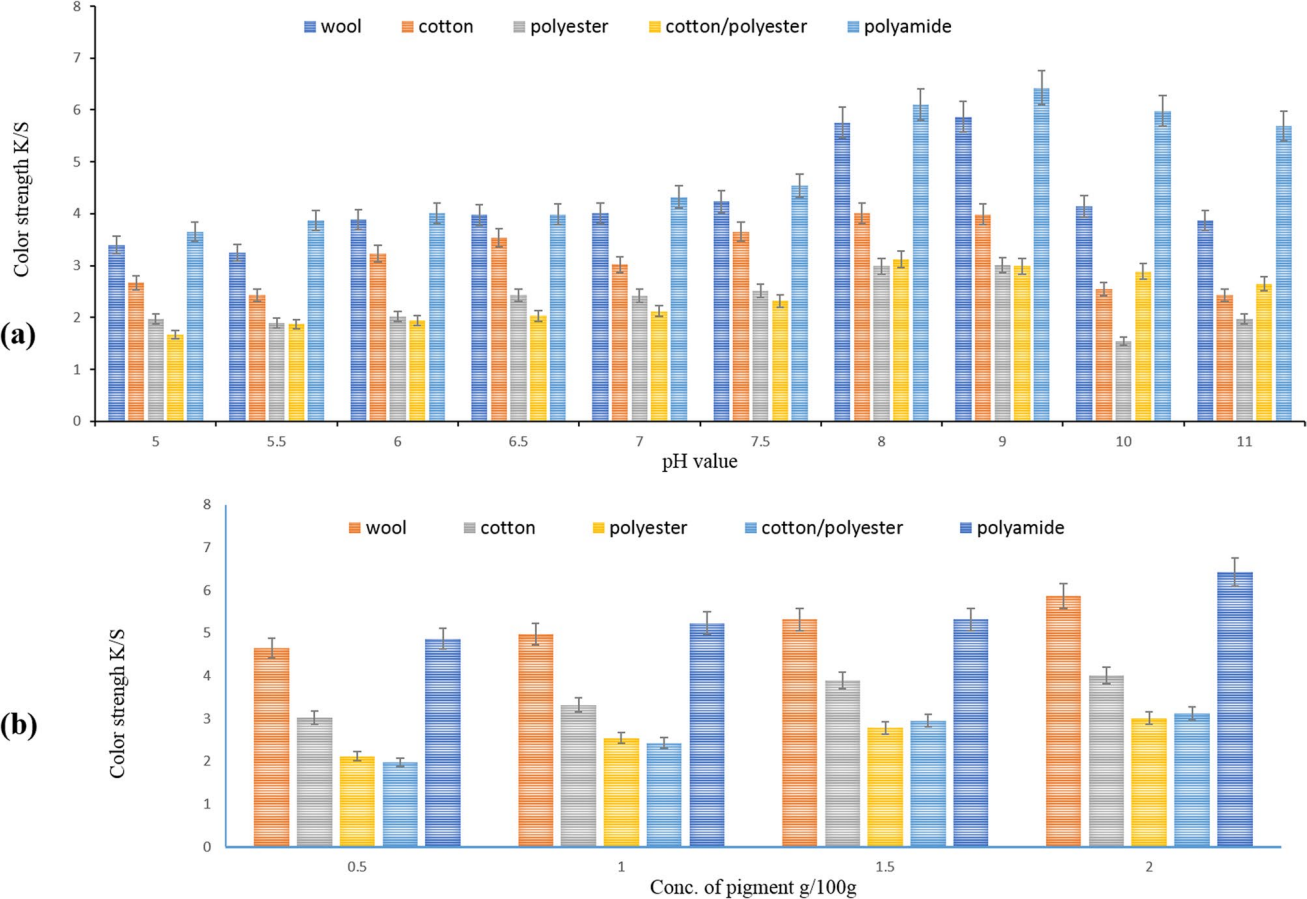
**a** Effect of pH, **b** Effect of concentration of pigment on the printing of cotton, wool, cotton/polyester, polyamide and polyester fabrics with brown pigment GH80 extracted from *S. erythrogriseus*. Data are expressed as Mean ± S.D. for n=3

The information you provided in Fig. 8(a), which illustrates the impact of pH on the dyeing of various fabrics using a brown pigment derived from *S. erythrogriseus* GH80. Let’s examine the findings for each fabric type at different pH to emphasize their unique characteristics and potential. Wool fabrics exhibit a significant increase in color intensity from pH 5 to pH 8, peaking impressively at a value of 5.76 at pH 8.

However, this intensity diminishes at pH 9 and 10, indicating that wool performs best within a near-neutral pH range (6–8) for optimal dye uptake and vibrant color development.

Cotton reveals an interesting trend, with a consistent rise in color intensity from pH 5 (2.67) to pH 6.5 (3.54), followed by stabilization at around 4.01 at pH 7. Notably, intensity decreases beyond pH 7, suggesting that slightly acidic to neutral conditions are ideal for effective dyeing of cotton. Conversely, polyester fabrics maintain relatively low color intensity through the pH range, indicating limited reactivity to the pigment. The highest observed value is 3.12 at pH 7.5, which remains modest compared to wool and cotton, highlighting the need for alternative methods for dyeing this fabric. The cotton/polyester blend shows promise with intermediate results, peaking at pH 7.5 (3.12). It still falls short of the effectiveness demonstrated by wool and cotton. The data suggests that the blend’s dye uptake is influenced by both fiber types, with optimal conditions likely centered around pH, offering an opportunity for refinement in blending techniques.

Polyamide with the highest color intensity values, peaking at pH 8 with a strong measurement of 6.43. This high affinity for pigment at elevated pH indicates that polyamide can effectively utilize this pigment for dynamic printing, making it an excellent choice for high-quality dye applications. So, each fabric type demonstrates distinct pH-dependent dyeing behavior, with wool, cotton, and polyamide showing more favorable uptake at near-neutral pH levels, while polyester remains less responsive overall [56].

### Effect of pigment concentration

Figure 8(b) clearly demonstrates that the concentration of brown pigment in the printing process plays a crucial role in enhancing the dye uptake and color strength (K/S values) of various fabrics, including wool, cotton/polyester, polyamide, and polyester. Our study reveals that increasing the dye concentration leads to a significant improvement in the color strength of dyed fabrics. Specifically, when the dye concentration is raised from 0.5% to 2% based on the fabric’s form, we observe a remarkable enhancement in K/S values across all fabric samples. This compelling evidence suggests that higher concentrations of brown pigment facilitate improved dye uptake and saturation within the fibers. Therefore, it is evident that to achieve optimal color strength for all tested fabrics, utilizing higher concentrations of brown pigment is essential, solidifying the necessity of reaching that saturation point at 2% [57].

### Color strength (K/S)

The effect of the brown pigment extracted from *S. erythrogriseus* GH80 on color strength varies significantly across different fabrics due to their unique fiber compositions and inherent properties. In Wool fabrics, are moderate to high L values suggest the pigment enhances depth without excessive darkening. Positive a and b values indicate a warmer color shift, potentially enriching the natural wool tones. A significant ΔE* value signifies a strong color impact. Higher K/S values suggest increased color strength and likely enhance the richness of the wool. Cotton L values are similar to wool, suggesting a vibrant shade without losing brightness. The pigment likely introduces a richer, warmer hue, as evidenced by changes in a and b. A considerable ΔE* value indicates effective pigment absorption and strong color performance. K/S values indicate a notable increase in color strength, improving the overall appearance. In polyester fabrics, the high L values indicate that the pigment retains brightness while adding color. The lower a and b values suggest a subtle alteration in color without a significant warming effect. A moderate ΔE* value indicates that there is effective interaction between the pigment and the polyester fibers. Additionally, K/S values may be lower than those of natural fibers because polyester has a lower absorbency. Cotton/polyester blend, very high L values may limit significant darkening. A negative b value indicates potential for a cooler tone. A minimal ΔE* value suggests limited color alteration due to the blend’s nature. K/S values are the lowest among the fabrics, indicating inherent limitations in achieving high color strength with this pigment on the blend. L values of polyamide are similar to wool, suggesting good pigment interaction. Positive color parameters indicate potential for enriching warmer shades. A strong ΔE* value shows effective pigment absorption. Moderate to high K/S values indicate good color strength enhancement [58].

In conclusion, the brown pigment enhances the color strength of various fabrics to different degrees. Natural fibers like wool and cotton demonstrate more significant improvements in color depth and strength compared to synthetic fibers like polyester, likely due to differences in their chemical structures. The cotton/polyester blend exhibits the least effect, highlighting the crucial role of fiber composition in pigment absorption and subsequent color strength enhancement [59] (Table 3).

**Table 3:**
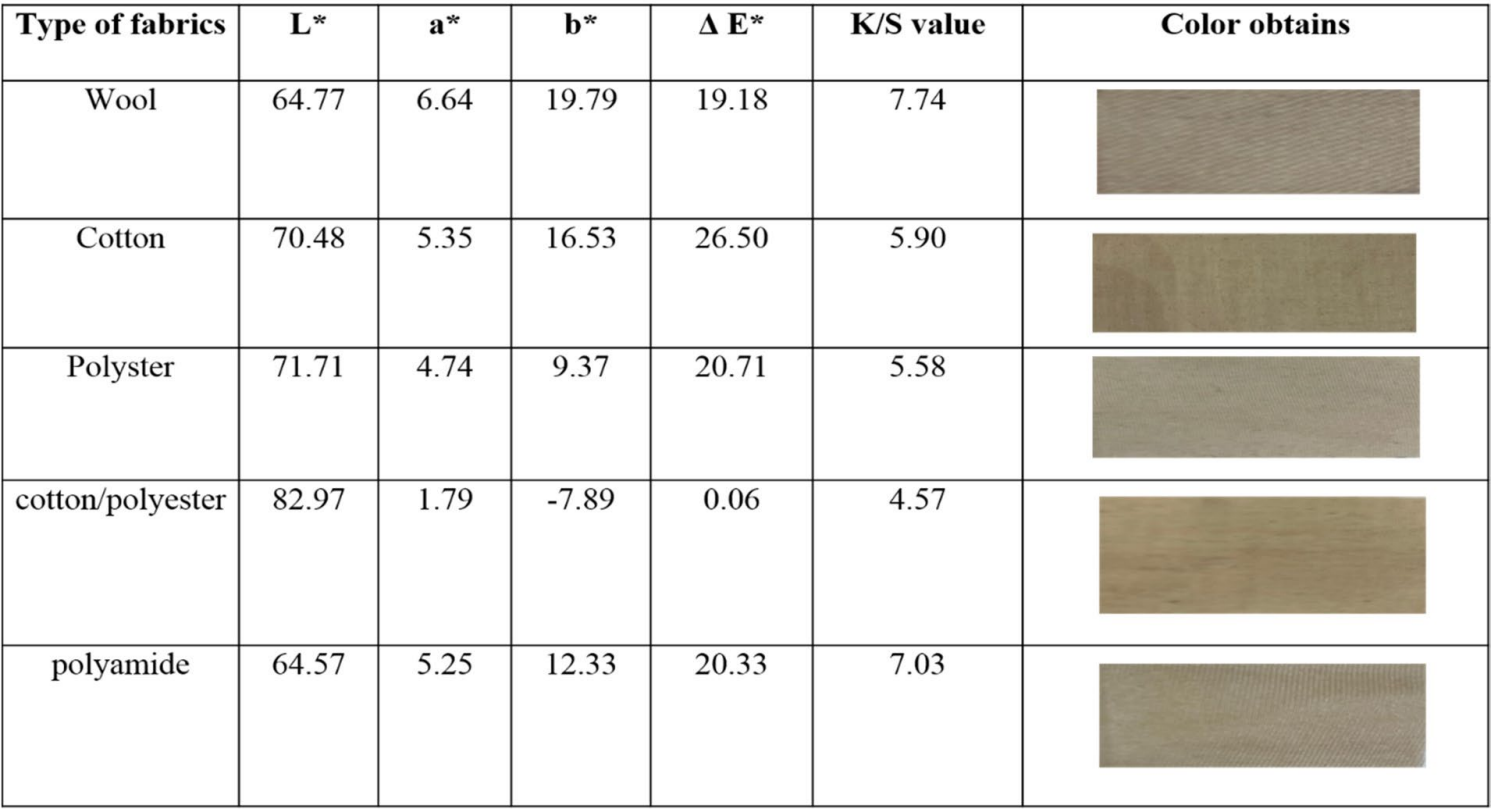
Color strengths and obtained colors of extracted brown pigment on the fabrics

### Self-Cleaning

The brown pigment GH80 significantly improves the self-cleaning properties of various fabrics, especially wool and cotton, when exposed to UV light. This indicates potential applications in textiles designed to enhance maintenance and durability. Further research could investigate the mechanisms of action and the long-term stability of the pigment on these fabrics [56].

Table 4 is shows the effectiveness of piment GH80 on the fabrics. Wool exhibited the highest % decrease in K/S (38%), indicating significant improvement in self-cleaning ability. Cotton followed closely with a 35% decrease, showing it also benefits considerably from the pigment. Polyester and the cotton/polyester blend had decreases of 32% and 30%, respectively, suggesting good performance but slightly less than wool and cotton alone. Polyamide had the least effect with a 24% decrease, indicating it may not respond as effectively to the pigment for self-cleaning purposes. The K/S value represents the color strength and is a measure of the amount of light absorbed by the fabric. A decrease in K/S indicates a reduction in color intensity, which can be associated with the breakdown of contaminants or the degradation of the dye.

**Table 4:** The effect of extracted brown pigment on the self-cleaning of natural, synthetic and blend fabrics

**Type of sample**	**K/S**		(%) decrease in K/S*
	Before exposure to UV*	After exposure to UV*	
Wool	7.74 ± 0.23	5.71 ± 0.17	38 ± 1.2
Cotton	5.90 ± 0.18	4.16 ± 0.13	35 ± 1.1
Polyester	5.58 ± 0.17	3.98 ± 0.12	32 ± 1.0
Cotton/polyester	4.57 ± 0.14	3.99 ± 0.12	30 ± 0.9
Polyamide	7.03 ± 0.21	6.87 ± 0.21	24 ± 0.8
UV exposure time 24 hours			

* Data are expressed as Mean ± S.D. for n=3

### UV protection

*S. erythrogriseus* GH80 produces a brown pigment that can serve as a natural dye. When applied to textiles such as cotton, wool, polyester, polyamide, and blends, this pigment can affect ultraviolet (UV) protection, or Ultraviolet Protection Factor (UPF), such as absorption of UV radiation, color intensity, and fabric composition [57].

*S. erythrogriseus* GH80 produces a brown pigment with potential for use as a natural dye. When applied to textiles (cotton, wool, polyester, polyamide, and blends), this pigment can influence ultraviolet (UV) protection, specifically UV absorption, color intensity, and ultimately, the Ultraviolet Protection Factor (UPF). The brown pigment can absorb UV radiation, potentially increasing the UPF of treated fabrics. Natural pigments often contain chromophores that absorb specific wavelengths of light, reducing UV penetration. Consequently, the depth of color achieved with the pigment plays a crucial role in its UV protective qualities. Darker and more intense pigments generally offer better UV protection compared to lighter shades.

Table 5 demonstrates that natural fibers like cotton may exhibit improved UV protection when dyed with the pigment. A maximum UPF of 57.34 was achieved at a pigment concentration of 2 g/l. However, it’s important to note that cotton inherently exhibits lower UV resistance compared to synthetic fibers like polyester and polyamide.

**Table 5:** The effect of different concentration of extracted brown pigment on the UPF of natural, synthetic and blend fabrics

Type of sample	UPF at different conc. of brown pigment GH80			
Concentration of pigment GH8	62.34 ± 1.87	77.34 ± 2.32	88.45 ± 2.65	89.43 ± 2.68
Wool	33.32 ± 1.00	43.23 ± 1.30	44.5 ± 1.34	57.34 ± 1.72
Cotton	71.34 ± 2.14	71.54 ± 2.15	82.78 ± 2.48	84.34 ± 2.53
Polyester	62.32 ± 1.87	53.24 ± 1.60	72.43 ± 2.17	73.48 ± 2.20
Cotton/polyester	85.34 ± 2.56	94.43 ± 2.83	95.87 ± 2.88	98.99 ± 2.97
Polyamide	62.34 ± 1.87	77.34 ± 2.32	88.45 ± 2.65	89.43 ± 2.68

* Data are expressed as Mean ± S.D. for n=3.

Wool fabrics demonstrate excellent inherent UV protection with a UPF of approximately 89.43. Incorporating this natural pigment into wool textiles could offer a dual benefit: enhancing both aesthetic appeal and UV resistance, making the fabric more suitable for outdoor applications.

Polyester, polyamide, and blended fabrics possess higher inherent UV resistance. When dyed with the brown pigment, their overall UV protection may further increase due to the pigment’s UV absorption properties. The final UPF will depend on the specific fiber composition and the effectiveness of the pigment in providing UV protection within that particular fabric.

### Fastness properties

The brown pigment exhibits varying fastness properties across different fabrics. As shown in Table 6, wool and polyamide demonstrate the best overall fastness, while cotton also performs well. Polyester shows limitations, particularly in washing and rubbing fastness. Cotton/polyester blends exhibit intermediate fastness properties, combining the attributes of both fiber types. The effectiveness of the pigment in enhancing fastness is significantly influenced by the inherent characteristics of each fabric. For wool, washing fastness is generally high (4–5), indicating excellent color retention. Rubbing fastness is good to excellent, particularly in dry conditions. Perspiration fastness is strong, demonstrating good resistance to both acidic and alkaline perspiration. Lightfastness is excellent (5–6), suggesting high resistance to fading from light exposure [60].

**Table 6:** Color strength (K/S) fastness properties of printed fabrics with different concentration of extracted brown pigment

Fabric samples	Washing fastness		Rubbing fastness		Perspiration fastness				Light fastness
					Acidic		Alkaline		
	St.	Alt.	Dry	Wet	St.	Alt.	St.	Alt.	
wool	4–5 ± 0.15	4–5 ± 0.15	4 ± 0.12	3–4 ± 0.11	4–5 ± 0.15	4–5 ± 0.15	4–5 ± 0.15	4–5 ± 0.15	5–6
	4–5 ± 0.15	5 ± 0.15	4–5 ± 0.15	4 ± 0.12	5 ± 0.15	4–5 ± 0.15	4–5 ± 0.15	5 ± 0.15	6
	5 ± 0.15	4–5 ± 0.15	4–5 ± 0.15	4 ± 0.12	4–5 ± 0.15	5 ± 0.15	4–5 ± 0.15	4–5 ± 0.15	5–6
	5 ± 0.15	5 ± 0.15	4 ± 0.12	4 ± 0.12	5 ± 0.15	4–5 ± 0.15	4–5 ± 0.15	5 ± 0.15	5–6
Cotton	4 ± 0.12	4 ± 0.12	3–4 ± 0.11	3–4 ± 0.11	4–5 ± 0.15	4 ± 0.12	4–5 ± 0.15	4–5 ± 0.15	5–6
	4–5 ± 0.15	4 ± 0.12	4 ± 0.12	4 ± 0.12	5 ± 0.15	4–5 ± 0.15	4–5 ± 0.15	5 ± 0.15	5
	4–5 ± 0.15	4–5 ± 0.15	4–5 ± 0.15	4–5 ± 0.15	4–5 ± 0.15	4–5 ± 0.15	5 ± 0.15	5 ± 0.15	5–6
	5 ± 0.15	5 ± 0.15	4–5 ± 0.15	4 ± 0.12	4–5 ± 0.15	5 ± 0.15	4–5 ± 0.15	4–5 ± 0.15	5
polyester	4 ± 0.12	3–4 ± 0.11	3–4 ± 0.11	3–4 ± 0.11	3–4 ± 0.11	3–4 ± 0.11	3–4 ± 0.11	4 ± 0.12	4–5
	3–4 ± 0.11	4 ± 0.12	4 ± 0.12	3–4 ± 0.11	4 ± 0.12	3–4 ± 0.11	3–4 ± 0.11	4 ± 0.12	4
	4 ± 0.12	3–4 ± 0.11	4 ± 0.12	4 ± 0.12	3–4 ± 0.11	4 ± 0.12	3–4 ± 0.11	3–4 ± 0.11	4–5
	4 ± 0.12	3–4 ± 0.11	3–4 ± 0.11	3–4 ± 0.11	3 ± 0.09	3–4 ± 0.11	4 ± 0.12	3–4 ± 0.11	4–5
Cotton/polyester	4–5 ± 0.15	4 ± 0.12	4 ± 0.12	3–4 ± 0.11	4 ± 0.12	3–4 ± 0.11	4 ± 0.12	4 ± 0.12	5
	4–5 ± 0.15	4–5 ± 0.15	3–4 ± 0.11	3 ± 0.09	4 ± 0.12	4 ± 0.12	3–4 ± 0.11	3–4 ± 0.11	4–5
	4 ± 0.12	4 ± 0.12	4 ± 0.12	3–4 ± 0.11	4 ± 0.12	3–4 ± 0.11	4 ± 0.12	3–4 ± 0.11	5
	4–5 ± 0.15	4–5 ± 0.15	4 ± 0.12	4 ± 0.12	3–4 ± 0.11	3–4 ± 0.11	4 ± 0.12	4 ± 0.12	4–5
polyamide	4–5 ± 0.15	4–5 ± 0.15	3–4 ± 0.11	3–4 ± 0.11	4–5 ± 0.15	5 ± 0.15	4–5 ± 0.15	4–5 ± 0.15	5–6
	4–5 ± 0.15	5 ± 0.15	4 ± 0.12	4 ± 0.12	4–5 ± 0.15	4–5 ± 0.15	4 ± 0.12	4–5 ± 0.15	5
	5 ± 0.15	4–5 ± 0.15	3–4 ± 0.11	3–4 ± 0.11	5 ± 0.15	4–5 ± 0.15	4–5 ± 0.15	4–5 ± 0.15	5–6
	4–5 ± 0.15	5 ± 0.15	4 ± 0.12	3–4 ± 0.11	4–5 ± 0.15	5 ± 0.15	4–5 ± 0.15	4 ± 0.12	5–6

* Data are expressed as Mean ± S.D. for n=3

For cotton, washing fastness is fairly good (4) with some variability between samples. Rubbing fastness is good to very good, especially in dry conditions. Perspiration fastness is moderate to good, indicating some sensitivity to perspiration. Lightfastness is generally high (5–6), maintaining good color stability under light exposure. For polyester, washing fastness is moderate (3–4), indicating some color loss during washing. Rubbing fastness is lower compared to other fibers, especially in wet conditions. Perspiration fastness is moderate, showing vulnerability to both acidic and alkaline perspiration. Light fastness is fair to good (4–5), which is typical for synthetic fibers. For cotton/polyester blends, washing fastness is generally good (4–5), indicating decent color retention. Rubbing fastness is moderate to good, reflecting the combined properties of the fibers. Perspiration fastness is fair, showing a mixed response due to the blend composition. Light fastness is good but may vary depending on the blend ratio. For polyamide, washing fastness is high (4–5), indicating good dye retention. Rubbing fastness is good, especially in dry conditions. Perspiration fastness is excellent, demonstrating strong resistance to both acidic and alkaline perspiration. Lightfastness is very good to excellent (5–6), indicating high resistance to fading from light exposure [61].

### Antibacterial properties of treated fabrics

**Fig. 9:**
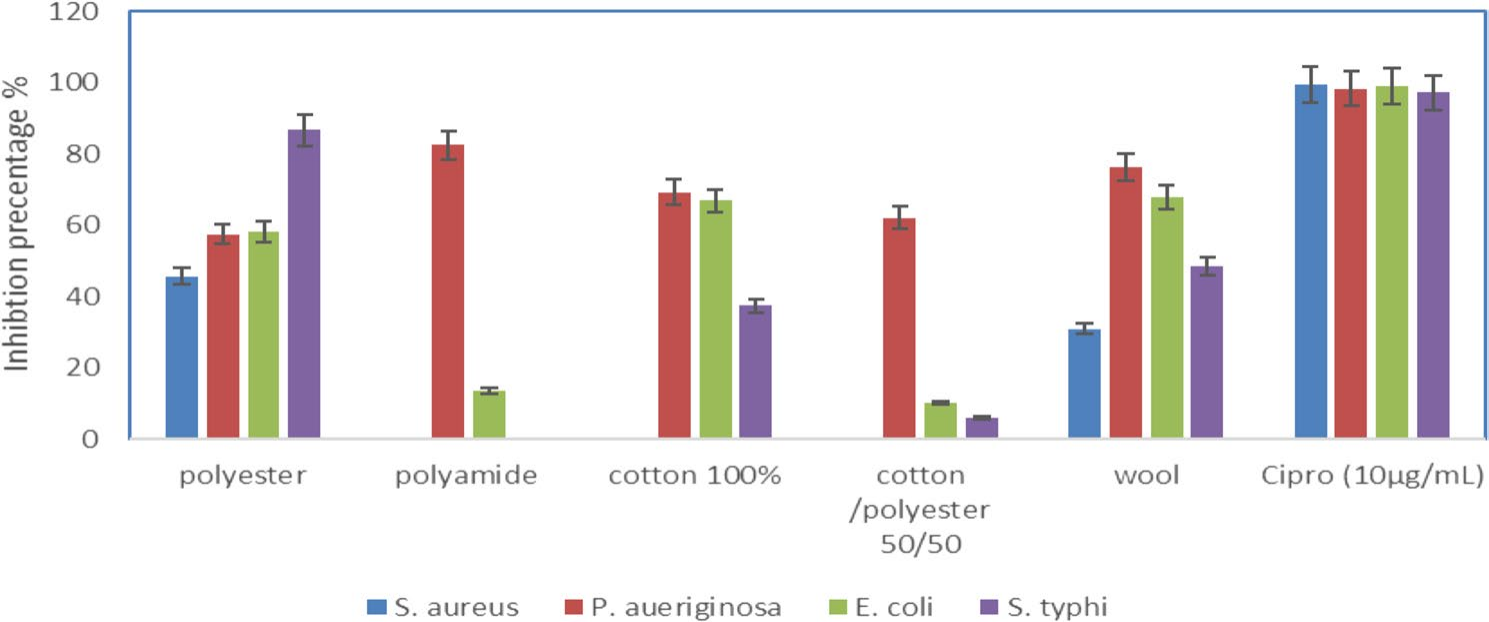
Antibacterial activity of treated fabrics. Data are expressed as Mean ± S.D. for n=3

The results indicate a differential inhibition of bacterial strains based on the type of textile treatment. *Salmonella typhi* showed the highest inhibition in polyester (86.6%). *Pseudomonas aeruginosa* showed the highest inhibition in polyamide (82.37). *Escherichia coli* and *Pseudomonas aeruginosa* showed strong inhibition in 100% cotton (67.9% and 76.16%, respectively). *Pseudomonas aeruginosa* had strong inhibition in cotton/polyester 50/50 (62.059). The control, ciprofloxacin (10 µg/mL), showed the highest inhibition for all strains, with *Staphylococcus aureus* reaching 99.25%. The effectiveness of textiles against different bacterial species varies significantly, with wool and polyester showing broader antimicrobial efficacy (Fig. 9). El Sayed et al. (2025) exhibited different levels of efficiency of treated cotton, wool, and polyester samples in inhibiting the growth of different pathogens [1].

### ADME-physiochemical properties of most abundant compound

The physicochemical profile of terbinafine reveals several noteworthy characteristics influencing its pharmacological behavior. With a molecular formula of C_21_H_25_N and a molecular weight of 291.43 g/mol, terbinafine comprises 22 heavy atoms, 10 of which are aromatic. It has a moderate amount of sp³ carbon (0.33%) and four bonds that can be rotated, which suggests that its structure is pretty flexible, which could make it easier for it to interact with biological targets. Notably, it has a single hydrogen bond acceptor and no donors, implying a limited capacity for hydrogen bonding, which could affect its solubility and permeability (Table 7).

**Table 7:** Physicochemical Properties

Physicochemical Properties	Value
Formula	C21H25N
Molecular Weight	291.43 g/mol
Number of Heavy Atoms	22
Number of Aromatic Heavy Atoms	10
Fraction Csp³	0.33
Number of Rotatable Bonds	4
Number of H-bond Acceptors	1
Number of H-bond Donors	0
Molar Refractivity	97.31
TPSA	3.24 Å²
Lipophilicity	
Log P (iLOGP)	4.15
Log P (XLOGP3)	5.59
Log P (WLOGP)	4.81
Log P (MLOGP)	4.89
Log P (SILICOS-IT)	5.16
Consensus Log P	4.92
Water Solubility	
Log S (ESOL)	−5.24
Solubility	1.67e-03 mg/ml; 5.74e-06 mol/l
Class	Moderately soluble
Log S (Ali)	−5.42
Solubility	1.11e-03 mg/ml; 3.80e-06 mol/l
Class	Moderately soluble
Log S (SILICOS-IT)	−5.98
Solubility	3.04e-04 mg/ml; 1.04e-06 mol/l
Class	Moderately soluble
Druglikeness	
Lipinski	Yes; 1 violation: MLOGP >4.15
Ghose	Yes
Veber	Yes
Egan	Yes
Muegge	No; 2 violations: XLOGP3 >5, Heteroatoms < 2
Bioavailability Score	0.55
Medicinal Chemistry	
PAINS	0 alert
Brenk	1 alert: triple_bond
Leadlikeness	No; 1 violation: XLOGP3 >3.5
Synthetic Accessibility	3.09

**Fig. 10:**
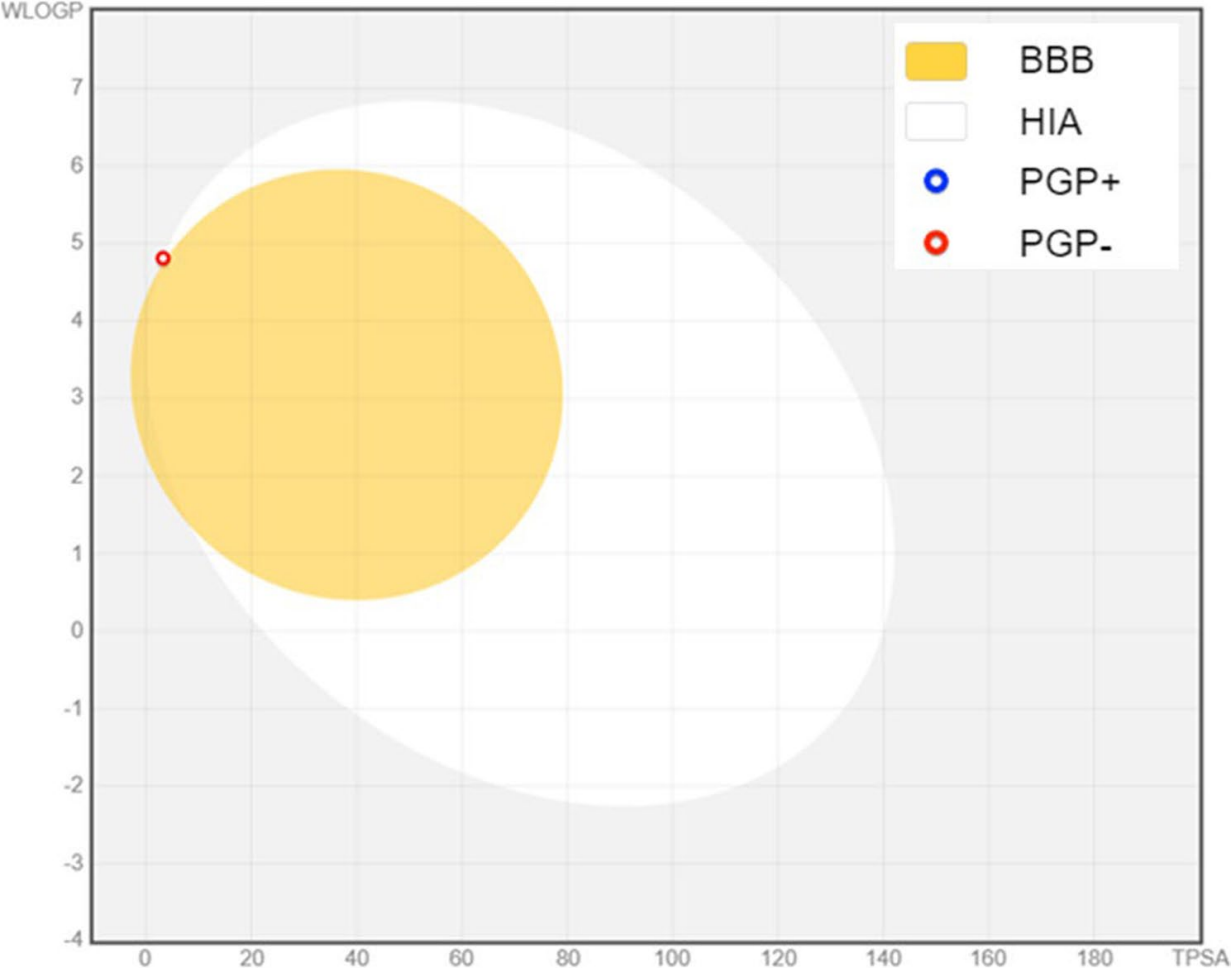
Boiled egg chart for Terbinafine

The lipophilicity of terbinafine is highlighted by its consensus log P values ranging from 4.15 to 5.59, indicating a tendency for high lipophilicity. This property, combined with a water solubility classified as moderately soluble (e.g., log S values around − 5.24), suggests that while terbinafine may effectively penetrate lipid membranes, its overall aqueous solubility is limited. Pharmacokinetic evaluations reveal high gastrointestinal absorption, although it does not permeate the blood-brain barrier (Fig. 10), which may be advantageous for targeting systemic fungal infections without central nervous system effects. Importantly, terbinafine acts as a CYP2D6 inhibitor, indicating potential drug interactions, while its synthetic accessibility score of 3.09 reflects moderate feasibility in its chemical synthesis (Table 8).

**Table 8:** Pharmacokinetics Properties

**Pharmacokinetics**	
GI Absorption	High
BBB Permeant	No
P-gp Substrate	No
CYP1A2 Inhibitor	No
CYP2C19 Inhibitor	No
CYP2C9 Inhibitor	No
CYP2D6 Inhibitor	Yes
CYP3A4 Inhibitor	No
Log Kp (Skin Permeation)	−4.11 cm/s

In terms of drug-likeness, terbinafine aligns with Lipinski’s rule, presenting one violation due to its log P exceeding 4.15. Its compliance with other parameters (Ghose, Veber, and Egan) underscores its viability as a medicinal candidate, despite a lack of ideal lead-likeness. Overall, the physicochemical attributes of terbinafine suggest a promising profile for antifungal activity, coupled with considerations for pharmacokinetic and drug interaction dynamics.

**Fig. 11:**
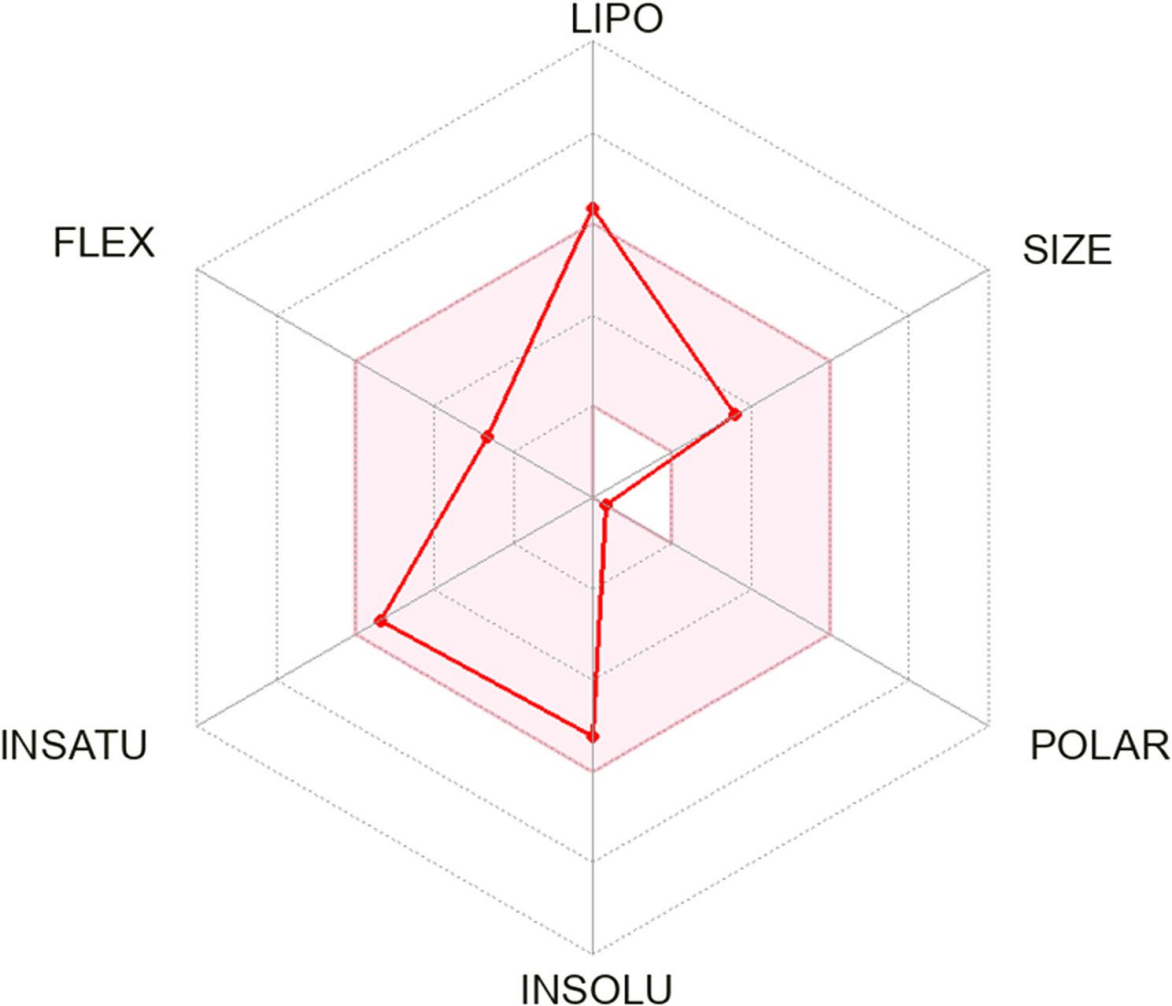
Bioavailability radar chart

The radar chart captures terbinafine’s bioavailability attributes by focusing on six pharmacokinetic factors: lipophilicity, size, polarity, insolubility, insaturation, and flexibility. Terbinafine exhibits a marked lipophilic nature, aligning with its strong attraction to lipid-dense tissues like skin and nails, where it performs its antifungal action. Its moderate levels of polarity and insolubility may pose challenges to dissolution in water-based environments, influencing its absorption. The balance between its molecular size and flexibility suggests an optimal structure for interaction with cellular membranes. Meanwhile, the lower unsaturation score indicates fewer double bonds, enhancing the compound’s chemical resilience. Overall, terbinafine’s strong lipid affinity boosts tissue penetration, though its moderate solubility might require adjustments in formulation for better absorption (Fig. 11).

### Toxicity prediction

The toxicity prediction for terbinafine, based on ProTox-3.0 analysis, reveals notable insights into its potential adverse effects across various biological systems. The compound shows a significant risk for hepatotoxicity and neurotoxicity, with probabilities of 0.66 and 0.69, respectively, indicating an active risk for these organ systems. Conversely, nephrotoxicity and cardiotoxicity appear less concerning, being classified as inactive with probabilities of 0.96 and 0.81, suggesting a lower likelihood of renal and cardiac adverse effects. Additionally, terbinafine poses a moderate risk for respiratory toxicity, with a high probability of 0.93.

**Fig. 12:**
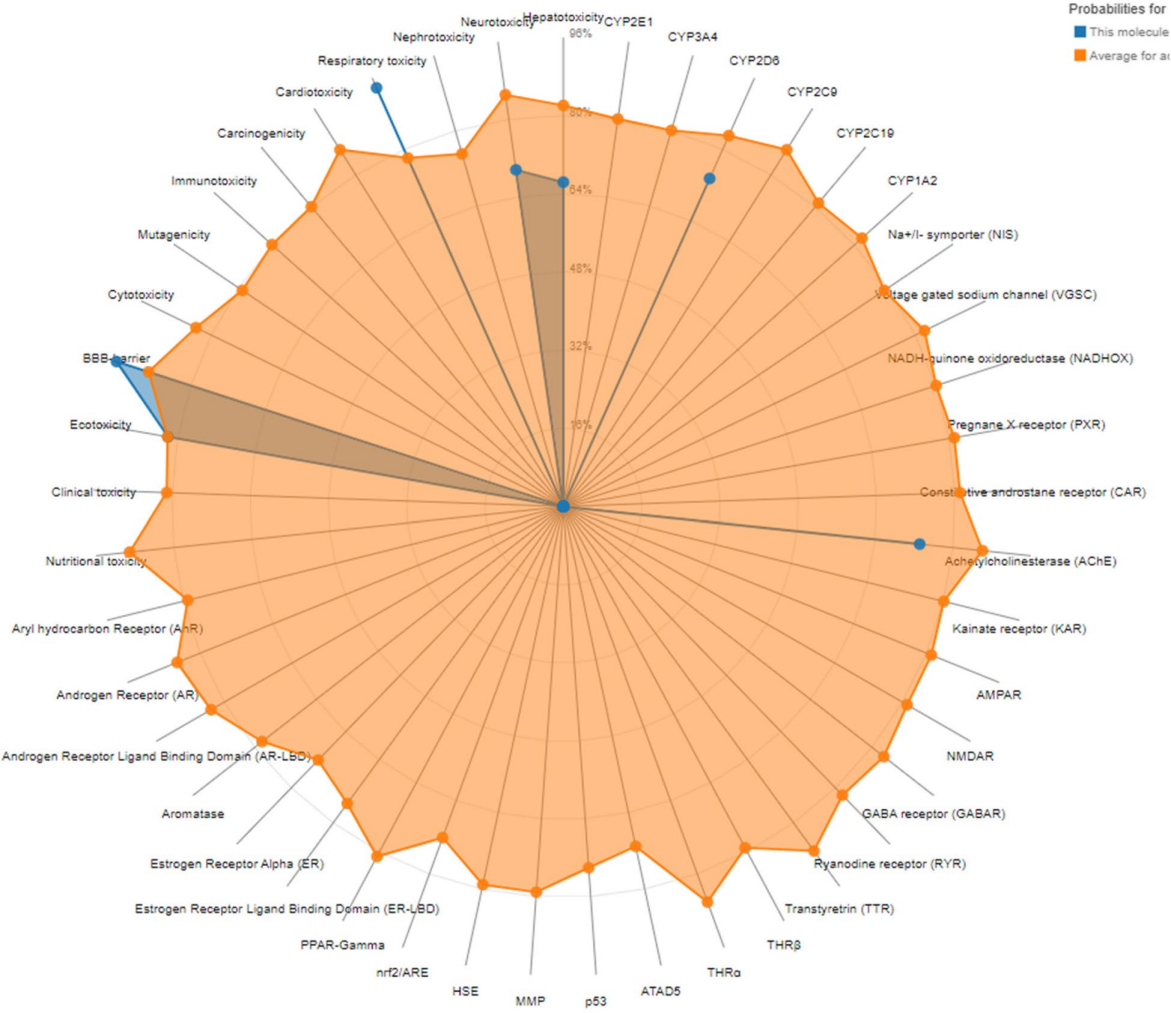
The toxicity radar chart is intended to quickly illustrate the confidence of positive toxicity results compared to the average of its class

In terms of broader toxicity endpoints, it is classified as inactive for carcinogenicity, immunotoxicity, mutagenicity, and cytotoxicity, which is favorable for its safety profile. However, it does show active status regarding blood-brain barrier penetration, with a high probability of 0.95, necessitating caution for potential central nervous system effects. Moreover, the analysis indicates inactivity across various nuclear receptor signaling pathways and stress response pathways, suggesting limited interactions with critical molecular initiating events that could lead to systemic toxicity. Notably, the acetylcholinesterase receptor is active with a probability of 0.73, indicating potential interactions that may influence neurochemical signaling. Overall, while terbinafine exhibits specific toxic risks, particularly to the liver and nervous system, its profile suggests a manageable safety margin concerning many common toxicity endpoints (Fig. 12).

## Conclusion

The pigment extract examined in this study exhibited a chemically diverse profile, with terbinafine identified as the primary component, in addition to several fatty acid methyl esters and minor metabolites. Terbinafine’s predominance underscores its potential contribution to the biological activity of the pigment, while the presence of additional bioactive compounds indicates possible synergistic effects. The extract, while primarily identified as a pigment, demonstrated an enrichment of pharmacologically significant metabolites, indicating its potential utility not only as a natural colorant but also as a source of antimicrobial and therapeutic agents. Additional research on pigment stability, comprehensive toxicological evaluations, and assessments of environmental safety is necessary to enhance its potential applications in industry and biomedicine.

### Statistical analysis

To make sure the experimental data was reliable and reproducible, statistical analysis was carried out by measuring the standard deviation (SD) of triplicate measurements.

## Supplementary Information

Supplementary material 1.

## Data Availability

The NCBI Gene Bank database accepted the Streptomyces isolate identified in the current study and registered it with accession number OQ345682.1. https://www.ncbi.nlm.nih.gov/search/all/?term=OQ1345682.1.%20.

## Abbreviations

*S*: *Streptomyces*
rpm: Rotation per minute
λmax: Maximum absorption wavelength
GC/MS: Gas chromatography-mass spectrometry
ATCC: American type culture collection strain
(NB): Nutritional broth
BLAST: Basic Local Alignment Search Tool
DMSO: Dimethyl sulfoxide
USA: The company's products are widely distributed, including in the USA.
Oxoid: A globally recognized brand of laboratory products, including culture media and diagnostic reagents, primarily for microbiology
UK: An abbreviation for the United Kingdom, a country
(SMF): Solid-state fermentation
(MK): Monacolin K, a compound known for its cholesterol-lowering properties
(K/S): Tinctorial strength (Color strength)
(AATCC): The American association of textile chemists and colorists
Conc.: Concentration
(UPF): UV protection factor
(AS/NZS): The Australian/New Zealand standard

## Notes

**Funding**.

Open access funding provided by the Science, Technology & Innovation Funding Authority (STDF) in cooperation with the Egyptian Knowledge Bank (EKB).
