# Supplementary material for: Functionalization of textile using streptomyces erythrogriseus GH80 brown bioactive pigment with in silico studies
Source: BMC Microbiol. 2025 Dec 17;26:42. doi: 10.1186/s12866-025-04503-5 (PMC12821193; doi:10.1186/s12866-025-04503-5)
Supplement: Supplementary file 1 — Supplementary material 1. [file 12866_2025_4503_MOESM1_ESM.docx]

Supplementary data presented in figure 8(a), the effect of pH on the printing paste

| Different pH | 5 | 5.5 | 6 | 6.5 | 7 | 7.5 | 8 | 9 | 10 | 11 |
| --- | --- | --- | --- | --- | --- | --- | --- | --- | --- | --- |
| wool | 3.4 | 3.25 | 3.89 | 3.98 | 4.01 | 4.23 | 5.76 | 5.87 | 4.14 | 3.87 |
| cotton | 2.67 | 2.43 | 3.23 | 3.54 | 3.02 | 3.65 | 4.01 | 3.99 | 2.54 | 2.43 |
| polyester | 1.97 | 1.89 | 2.021 | 2.43 | 2.42 | 2.51 | 2.99 | 3.01 | 1.54 | 1.97 |
| cotton/polyester | 1.67 | 1.87 | 1.94 | 2.031 | 2.12 | 2.32 | 3.12 | 2.99 | 2.89 | 2.65 |
| polyamide | 3.65 | 3.87 | 4.01 | 3.99 | 4.32 | 4.54 | 6.1 | 6.43 | 5.98 | 5.69 |
